# Alcohol sedation in adult *Drosophila* is regulated by *Cysteine proteinase-1* in cortex glia

**DOI:** 10.1038/s42003-019-0492-5

**Published:** 2019-07-03

**Authors:** Kristen M. Lee, Laura D. Mathies, Mike Grotewiel

**Affiliations:** 1 Neuroscience Graduate Program, Virginia Commonwealth University, Richmond, VA 23298 USA; 2 Department of Pharmacology and Toxicology, Virginia Commonwealth University, Richmond, VA 23298 USA; 3 Virginia Commonwealth University Alcohol Research Center, Virginia Commonwealth University, Richmond, VA 23298 USA; 4 Department of Human and Molecular Genetics, Virginia Commonwealth University, Richmond, VA 23298 USA

**Keywords:** Glial biology, Behavioural genetics, Glial biology, Behavioural genetics

## Abstract

Although numerous studies have demonstrated that neuronal mechanisms regulate alcohol-related behaviors, very few have investigated the direct role of glia in behavioral responses to alcohol. The results described here begin to fill this gap in the alcohol behavior and gliobiology fields. Since *Drosophila* exhibit conserved behavioral responses to alcohol and their CNS glia are similar to mammalian CNS glia, we used *Drosophila* to begin exploring the role of glia in alcohol behavior. We found that knockdown of *Cysteine proteinase-1* (*Cp1*) in glia increased *Drosophila* alcohol sedation and that this effect was specific to cortex glia and adulthood. These data implicate *Cp1* and cortex glia in alcohol-related behaviors. Cortex glia are functionally homologous to mammalian astrocytes and Cp1 is orthologous to mammalian Cathepsin L. Our studies raise the possibility that cathepsins may influence behavioral responses to alcohol in mammals via roles in astrocytes.

## Abstract (web summary)

Kristen Lee et al. show that knockdown of *Cysteine proteinase-1* (*Cp1*) in glia increases sedation of adult *Drosophila* in response to alcohol. This study suggests a possibility that mammalian Cp1 homologue cathepsins in astrocytes may influence behavioral responses to alcohol.

## Introduction

Alcohol use disorder, defined as chronic alcohol abuse and dependence (DSM-V)^1^, is a major health problem. For example, alcohol abuse is the third leading risk factor for death and disability^2^, excessive alcohol consumption is estimated to be responsible for ~2.5 million preventable deaths worldwide per year, and alcohol abuse costs the United States ~200 billion dollars annually^3–5^. Thus, there is a pressing need to better understand the mechanisms involved in the development of alcohol use disorder, identify individuals at risk for alcohol use disorder, and ultimately provide improved treatment options for the disorder.

In largely naive alcohol drinkers, the initial level of response to alcohol correlates with their likelihood of becoming alcohol dependent^6^, a phenotype associated with alcohol use disorder^1^. For example, men with an initially low sensitivity to alcohol are four times more likely to be an alcoholic by age 30^6^. Therefore, investigating molecular-genetic mechanisms that influence alcohol sensitivity is a potentially promising approach for understanding the molecular underpinnings of alcohol use disorder.

The fruit fly *Drosophila melanogaster*, the nematode *C. elegans* and rodents have been used extensively to investigate the genetics of alcohol-related behaviors, including alcohol sedation. Numerous genes involved in alcohol-related behaviors in model organisms have human orthologs that have been implicated in human alcohol abuse, suggesting mechanistic connections between alcohol-related behaviors in model organisms and alcohol abuse in humans^7,8^. A majority of these genes are known or predicted to function in neurons^7^, leaving the contribution of glia and glial cell mechanisms to alcohol-related behavior largely unexplored. To the best of our knowledge, only three studies have investigated the direct contribution of glia in alcohol-related behaviors. One study found that activation of calcium signaling in rat nucleus accumbens core astrocytes via DREADDS decreases motivation for alcohol after a 3-week-long alcohol abstenance^9^. Another study found that *Drosophila* with a mutation in the gene *moody*, a gene expresssed in surface glia as well as other cell types, have reduced sensitivity to ethanol-induced loss of postural control^10^. An additional study in *Drosophila* found that surface glia also contribute to alcohol tolerance^11^. Despite these pioneering studies, our understanding of the role of glia in alcohol-related behavior is woefully incomplete.

The *Drosophila* central nervous system (CNS) is compartmentalized into two gross anatomical regions: an outer cortex (containing neuronal cell bodies) and a more central neuropil (containing neurites and synapses). Like mammals, the *Drosophila* CNS is composed of both neurons and glia. *Drosophila* CNS glia are functionally and molecularly similar to mammalian CNS glia^12–16^. Cortex glia, astrocytes, and ensheathing cells are the main subtypes of CNS glia in adult flies^12^. Additionally, perineural and subperineural glia, often referred to as surface glia, surround the entire CNS and compose the blood brain barrier in flies^17,18^. *Drosophila* cortex glia and astrocytes are intimately associated with neurons in the CNS^19^. Cortex glia are located in the cortex region of the brain and encapsulate virtually all neuronal cell bodies with fine processes^20^. A single adult cortex glial cell is thought to be able to encapsulate up to 100 neurons^19^. Cortex glia aid in gas exchange, neuronal firing, and nutrient transfer to neurons, similarly to mammalian protoplasmic astrocytes^12,20,21^. Cortex glia also exhibit calcium transients near membranes close to neurons, which appear to regulate neuronal cell function^22^. Physical associations between cortex glia and neurons are essential for normal nervous system function and behavior in *Drosophila*^23^. In contrast to cortex glia, the cell bodies of astrocytes reside at the cortex-neuropil interface and extend processes into the neuropil^24^. Like mammalian astrocytes, *Drosophila* astrocytes are important for synapse formation and maintenance, clearing and recycling neurotransmitters from the synapse, and modulating neuronal physiology^25,26^. *Drosophila* astrocytes release gliotransmitters, which are regulated by transient intracellular calcium signaling; this mechanism can directly influence nearby cells and influence behavior^15,27,28^. The cell bodies of ensheathing glia are also located at the interface of the brain cortex and neuropil^24^. Under normal physiological conditions, ensheathing glia encase the entire neuropil region in the CNS and occasionally wrap axonal segments between the neuropil and the periphery^29^. Ensheathing glia can regulate neuronal excitability by metabolizing glutamate, and disruptions in this function can alter behavior^30^. Under pathological conditions, these cells extend processes into the neuropil to phagocytize debris^24,31,32^. *Drosophila* surface glia (i.e., subperineural and perineural glia) are less similar to mammalian glia, but they have been associated with alcohol-related behavior in flies^10,11^. Subperineural glia mediate most of the blood brain barrier chemoprotective functions, similar to mammalian brain vascular endothelial cells^33^. Interestingly, subperineural glia can extend processes, which function at PNS synapses^34^. As their name implies, perineural glia reside on top of the subperineural glia, and protect against the entrance of larger molecules^35^. With macrophages, these cells secrete a dense lamella that covers the CNS and peripheral nerves^35^. Despite being extensively investigated in numerous experimental settings, a role for glia in fly alcohol-related behavior has not been comprehensively explored.

Here, we demonstrate that RNAi-mediated knockdown and rescue of the gene *Cysteine proteinase-1* (*Cp1*) constitutively in all CNS glia regulates alcohol sedation. This behavioral effect appears specific to *Cp1* expression in cortex glia, as well as all glia during adulthood. Cp1 is a hydrolase involved in protein degradation that is functionally and structurally homologous to mammalian Cathepsin L^36^. Our data suggest a previously unidentified role for cortex glia and *Cp1* in the adult *Drosophila* CNS: regulation of sedation in response to acute administration of alcohol.

## Results

### Identifying glial genes that influence alcohol sedation

To begin exploring the role of central nervous system (CNS) glia in alcohol behavior, we performed a targeted screen in which we compiled genes previously reported to be expressed in glia^13,37–39^, obtained genetic reagents to manipulate the expression of those genes, and determined whether constitutive or induced overexpression, expression of dominant negatives, or expression of RNAi targeting those genes influenced alcohol sedation. In total, we screened 19 genes by RNAi, nine by overexpression and five by dominant negatives.

One of the genes identified by this targeted screen was *Cysteine proteinase-1* (*Cp1*). *Cp1* is known to function in *Drosophila* midgut, garland cells, salivary glands, macrophages, gonads, and PNS neurons^36,40–43^ and is expressed in glia^13^, but prior to our results no studies have demonstrated a functional role for *Cp1* in glia. Cp1 is the only *Drosophila* cysteine proteinase that has been described and is functionally and structurally homologous to mammalian Cathepsin L^41,44^. Although cysteine proteinases play key roles in the lysosomes of phagocytic cells^43^ and mammalian Cathepsin L has been associated with multiple diseases, including cancer^45,46^, Alzheimer disease^47^, and retinal degeneration^41^, no previous studies implicate this family of genes in alcohol-related behavior.

### Glial *Cp1* regulates the pharmacodynamics of alcohol sedation

**Fig. 1:**
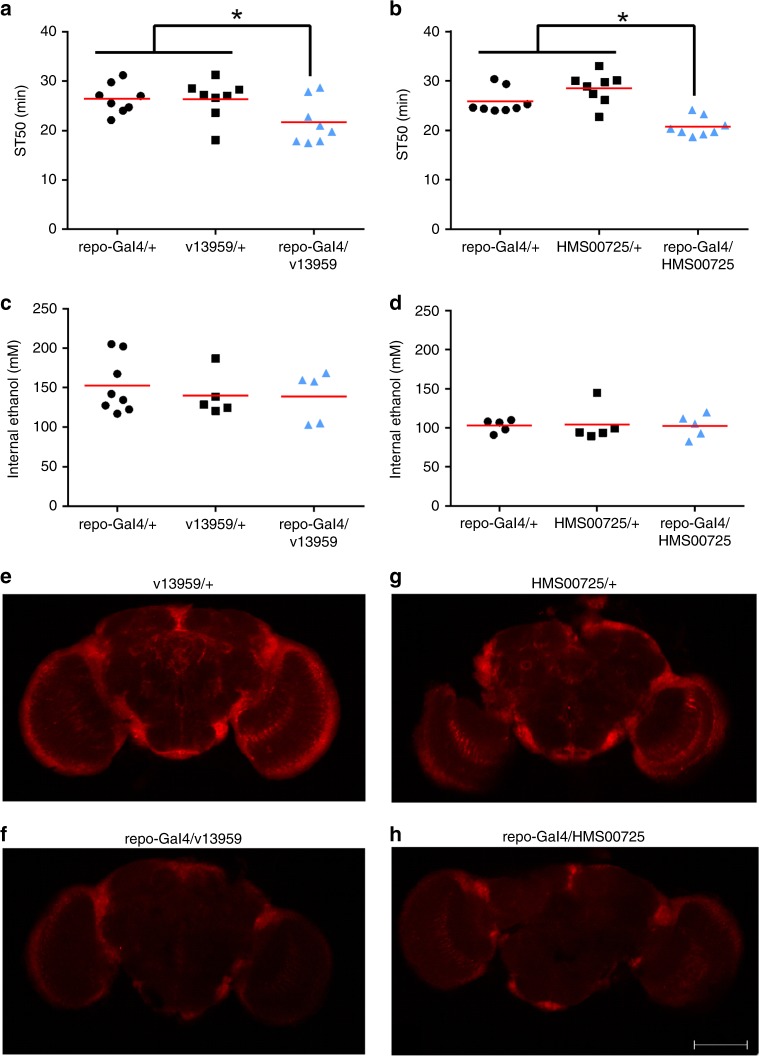
*Cp1* knockdown in CNS glia alters ethanol sedation sensitivity without affecting internal ethanol levels. **a**, **b** ST50 values were reduced in flies expressing *Cp1* RNAi transgenes in glia (blue bars: *repo*-Gal4/v13959, panel **a**; *repo*-Gal4/HMS00725, panel **b**) compared to control flies with either *repo*-Gal4 alone (black bars: *repo*-Gal4/+) or the RNAi transgenes alone (black bars: v13959/+ and HMS00725/+) (panel **a**: one-way ANOVA, *p* = 0.0352; *Bonferroni’s multiple comparison vs. controls, *p* < 0.05; *n* = 8; panel **b**: one-way ANOVA, *p* < 0.0001; *Bonferroni’s multiple comparison vs. control, *p* < 0.05; *n* = 8). **c**, **d** Expression of *Cp1* RNAi transgenes in CNS glia (blue bars: v13959, panel **c**; HMS00725, panel **d**) did not alter internal ethanol levels compared to controls with either *repo*-Gal4 or the RNAi transgenes alone (black bars) (individual one-way ANOVAs, *p* > 0.05; *n* = 8). **e**–**h** Whole-mount brain images immunolabeled for Cp1 expression (*n* = 5). Whole-brain Cp1 detection was reduced in flies expressing *Cp1* RNAi transgenes in glia (**f**, **h**) compared to brains from RNAi transgene control animals (**e**, **g**). (Anti-Cp1 1:250, Alexa 568 1:1000). Representative images, ×10

Flies with pan-glial Gal4 (*repo*-Gal4) driven expression of two different *Cp1* RNAi transgenes (v13959 and HMS00725, tested individually) had decreased sedation time 50 (ST50) values compared to control flies containing the Gal4 or an RNAi transgene alone (Fig. 1a, b). For reasons that are unclear, constitutive expression of a third RNAi transgene (v110619) in all glia did not consistently alter alcohol sedation (Supplementary Fig. 1a). To determine if *Cp1* influenced alcohol metabolism, we measured the internal alcohol levels in these same genotypes after a 30-min alcohol exposure (approximating the ST50). We found no significant difference in the internal alcohol concentrations between flies expressing *Cp1* RNAi transgenes in glia compared to controls (Fig. 1c, d), indicating that *Cp1* might influence a pharmacodynamic mechanism that impinges on alcohol sedation. Interestingly, despite *Cp1* being endogenously expressed in neurons^42^, pan-neuronal expression (via *elav*-Gal4) of a *Cp1* RNAi transgene (v13959) did not alter ST50 values compared to Gal4 and RNAi transgene controls (Supplementary Fig. 2a). Taken together, these results suggest that *Cp1* influences alcohol sedation via a role in glia. Although our studies are consistent with the hypothesis that *Cp1* function in neurons might not play a major role in alcohol sedation, further studies would be required to formally assess this possibility.

The principal RNAi transgenes used in this study (v13959 and HMS00725) are predicted to target all four mRNA transcripts of *Cp1* (Supplementary Fig. 3) and have no predicted off-target effects^48–50^. We used whole-brain immunofluorescence to address whether the RNAi transgenes knockdown Cp1 expression in specific tissues. Overall Cp1 immunofluorescence was substantially reduced (v13959: 55%; HMS00725: 62%) in brains from flies with pan-glial expression of *Cp1* RNAi transgenes (Fig. 1f, h) compared to brains from flies with the *Cp1* RNAi transgenes alone (Fig. 1e, g). The remaining Cp1 immunofluorescence is consistent with Cp1 expression in neurons, which should not be impacted by expression of *Cp1* RNAi in glia. Additionally, overall Cp1 immunofluorescence was reduced 29% in brains expressing the v13959 *Cp1* RNAi transgene pan-neuronally (Supplementary Fig. 2c) compared to brains containing the v13959 *Cp1* RNAi transgene alone (Supplementary Fig. 2b). The remaining Cp1 immunofluorescence is consistent with Cp1 expression in glia. These results confirm that expression of the *Cp1* RNAi transgenes knocked down *Cp1* as expected in both glia and neurons.

### Orthologous *Cp1* rescues *Cp1* knockdown

When expressed in glia, both of the main *Cp1* RNAi transgenes used in our studies (v13959 and HMS00725) make flies sensitive to alcohol sedation and knockdown *Cp1* expression (Fig. 1). The target sequence of HMS00725 is wholly encompassed by that of v13959 (Supplementary Fig. 3), raising the possibility that the sensitivity to alcohol sedation in flies expressing *Cp1* RNAi might be due to knockdown of *Cp1* or another, unidentified, gene. To address this possibility, we determined whether expression of a *Cp1* ortholog from *Drosophila pseudoobscura* in glia could rescue the alcohol sedation sensitivity in flies expressing RNAi against endogenous melanogaster *Cp1* also in glia^51^. We choose the *Drosophila pseudoobscura Cp1* ortholog (*GA25021*) for these studies because its primary amino acid sequence is 70–92 % similar to the four *Drosophila melanogaster Cp1* isoforms and the HMS00725 siRNA target sequence is poorly conserved between *Cp1* and* GA25021*—there are six base pair mismatches (Supplementary Fig. 4). Taken together, these findings suggested that GA25021 protein would have a similar function to *melanogaster* Cp1, but importantly the *GA25021* mRNA would largely escape RNAi-mediated degradation by HMS00725. We therefore postulated that expression of *GA25021* might rescue the alcohol sedation sensitivity observed in flies expressing RNAi against melanogaster *Cp1* in glia.

**Fig. 2:**
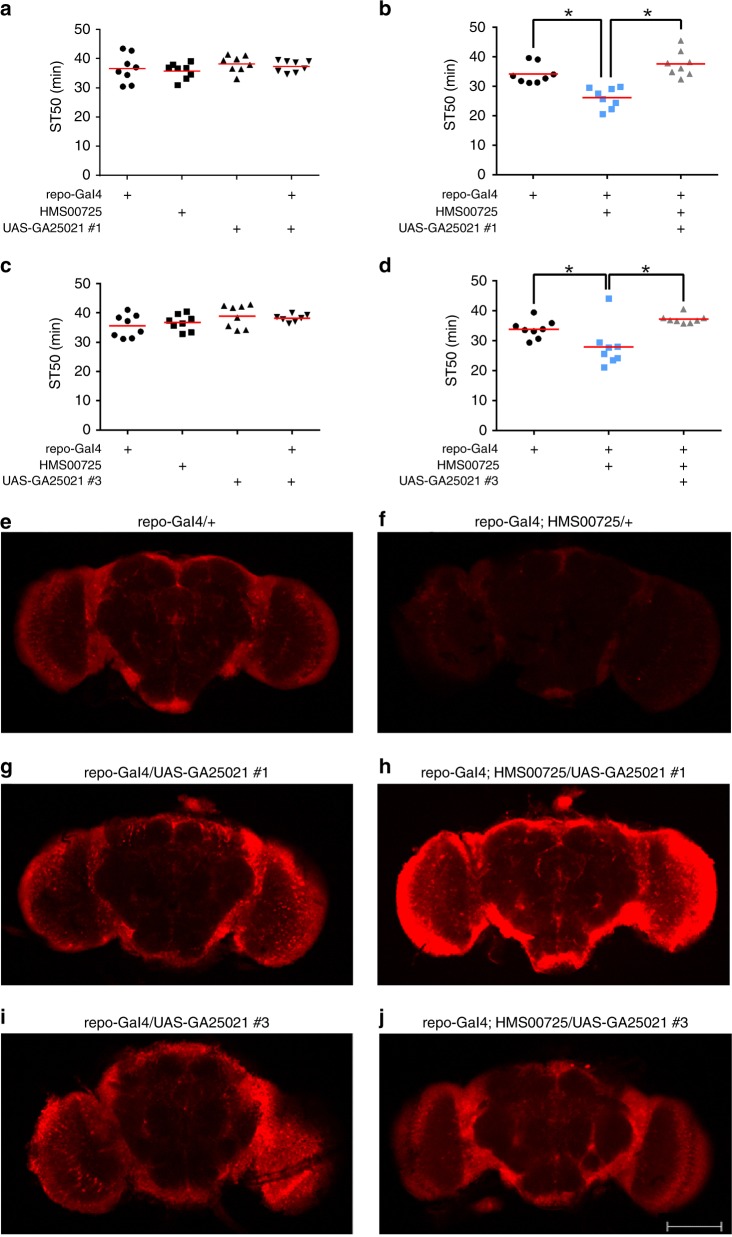
Trans-species rescue of alcohol sedation in *Cp1* RNAi flies. **a**, **c** Ethanol sedation in flies with *repo*-Gal4 alone, HMS00725 alone, UAS-*GA25021* transgenes alone, and *repo*-Gal4 with UAS-*GA25021*. Genotype did not impact ST50 values (panel **a**: one-way ANOVA, *p* = 0.4855, *n* = 8; panel **c**: one-way ANOVA, *p* = 0.1683, *n* = 8). **b**, **d** Ethanol sedation in flies with concurrent expression of *Cp1* RNAi and *GA25021*. ST50 values were decreased in flies constitutively expressing the HMS00725 *Cp1* RNAi transgene in all glia via *repo*-Gal4 (blue squares) compared to control flies containing *repo*-Gal4 alone (black circles). ST50 values in flies that expressed a UAS-*GA25021* transgene and HMS00725 *Cp1* RNAi in all glia via *repo*-Gal4 (gray triangles: UAS-*GA25021* #1, panel **b**; UAS-*GA25021* #3, panel **d**) were significantly elevated compared to flies expressing HMS00725 alone (blue squares: UAS-*GA25021* #1, panel **b**; UAS-*GA25021* #3, panel **d**), but were not different than control flies containing *repo*-Gal4 alone (black circles) (panel **b**: one-way ANOVA, *p* < 0.0001, *n* = 8,; panel **d**: one-way ANOVA, *p* = 0.0019; *Bonferroni’s multiple comparison vs. *repo*-Gal4;HMS00725 flies, *p* < 0.05). **e**–**j** Whole-mount brain images immunolabeled for Cp1. Whole-brain fluorescence was reduced in flies constitutively expressing the HMS00725 *Cp1* RNAi transgene in all glia via *repo*-Gal4 (**f**) compared to brains that contained *repo*-Gal4 alone (**e**). Compared to brains that contained *repo*-Gal4 alone (**e**), whole-brain fluorescence was increased when a UAS-*GA25021* transgene was expressed in all glia via *repo*-Gal4 (UAS-*GA25021* #1, panel **g**; UAS-*GA25021* #3, panel **i**). Compared to brains that expressed the HMS00725 *Cp1* RNAi transgene in all glia via *repo*-Gal4 (**f**), whole-brain fluorescence was increased when a UAS-*GA25021* transgene was expressed with the HMS00725 *Cp1* RNAi transgene in all glia via *repo*-Gal4 (UAS-*GA25021* #1, panel **h**; UAS-*GA25021* #3, panel **j**). Representative images from middle sections of adult brains, ×10 (Anti-Cp1 1:250; Alexa 568 1:1000)

We generated UAS-*GA25021* transgenic flies via P-element transgenesis and then selected six lines with transgenes that didn’t impact ST50 values in the absence or presence of *repo*-Gal4 (see Fig. 2a, c). We then assessed whether expression of these UAS-*GA25021* transgenes rescued alcohol sensitivity in constitutive glial *Cp1* knockdown flies. *repo-*Gal4/+ flies were used as a representative control in our subsequent rescue experiments because their ST50 values were not significantly different from other control flies that had the RNAi transgene alone, the UAS-*GA25021* transgene alone, or *repo*-Gal4 driven expression of a UAS-*GA25021* transgene (Fig. 2a, c).

Consistent with the data in Fig. 1b, flies that constitutively expressed the *Cp1* RNAi transgene HMS00725 in all glia (via *repo*-Gal4) had decreased ST50 values compared to control flies with *repo*-Gal4 alone (Fig. 2b, d). In contrast, flies with pan-glial expression of both the *Cp1* RNAi transgene HMS00725 and a UAS-*GA25021* transgene had increased ST50 values compared to flies expressing only the *Cp1* RNAi transgene HMS00725 and statistically indistinguishable ST50 values compared to control flies with *repo*-Gal4 alone (Fig. 2b, d). In total, we tested six UAS-*GA25021* transformants. The transgenes in four of the transformants rescued the glial *Cp1* RNAi alcohol sedation phenotype (including those in Fig. 2), while two of the transgenes did not. The ability of *Drosophila pseudoobscura* *Cp1* to rescue ethanol sedation sensitivity due to knockdown of *melanogaster* *Cp1* strongly supports a role for *Cp1* in glia in ethanol sedation.

We used whole-brain immunofluorescence to address whether the UAS-*GA25021* transgenes expressed detectable levels of immunoreactive Cp1-like protein. Endogenous Cp1 was readily detectable in control *repo*-Gal4/+ brains (Fig. 2e). This signal was reduced substantially by expression of HMS00725 *Cp1* RNAi in all glia (Fig. 2f; decreased 68%) and increased by expression of UAS-*GA25021* transgene #1 in all glia (Fig. 2g; increased 37%). Expression of this same UAS-*GA25021* transgene concurrently with HMS00725 substantially increased the Cp1 signal compared to brains that expressed only HMS00725 in all glia (Fig. 2h; increased 331%). Similarly, expression of UAS-*GA25021* transgene #3 in all glia increased the Cp1 signal (Fig. 2i; increased 32% compared to *repo*-Gal4 alone) and expression of this same UAS-*GA25021* transgene concurrently with HMS00725 substantially increased the Cp1 signal compared to brains that expressed only HMS00725 in all glia (Fig. 2e; 188%). Although we were surprised by—and do not at this time understand—the difference in Cp1 signal in flies with concurrent expression of *GA25021* and HMS00725, these data indicate that the UAS-*GA25021* transgenes are functional at the protein expression level. Additionally, we used real-time PCR to assess *Cp1* and *GA25021* expression in these studies (Supplementary Fig. 5). As expected, using primers for *Cp1* we readily detected a product in cDNA samples derived from control *repo*-Gal4/+ and from* repo*-Gal4/UAS-*GA25021* flies, and detection of this product was significantly reduced in flies expressing the HMS00725 *Cp1* RNAi alone in glia or in combination with UAS-*GA25021* (Supplementary Fig. 5, blue symbols). Also as expected, using primers for *GA25021* we readily detected a product in *repo*-Gal4/UAS-*GA25021* flies without or with concurrent expression of the *Cp1* RNAi HMS00725, and this product was not detectable in *repo*-Gal4/+ or *repo*-Gal4/HMS00725 flies (Supplementary Fig. 5, red symbols). These data confirm that the *Cp1* and *GA25021* primers are specific for each product, that the *Cp1* RNAi HMS00725 knocked down *Cp1* with or without *GA25021* expression, and that *GA25021* is expressed with or without expression of *Cp1* RNAi. The most parsimonious interpretation of the data in Fig. 2 and Supplementary Fig. 5 is that flies expressing the *Cp1* RNAi HMS000725 transgene and an orthologous gene via the UAS-*GA25021* transgene in glia have decreased Cp1 levels while expressing *GA25021*, thereby leading to the rescue of the behavioral phenotype due to *Cp1* knockdown in glia.

### *Cp1* expression in cortex glia regulates alcohol sedation

**Fig. 3:**
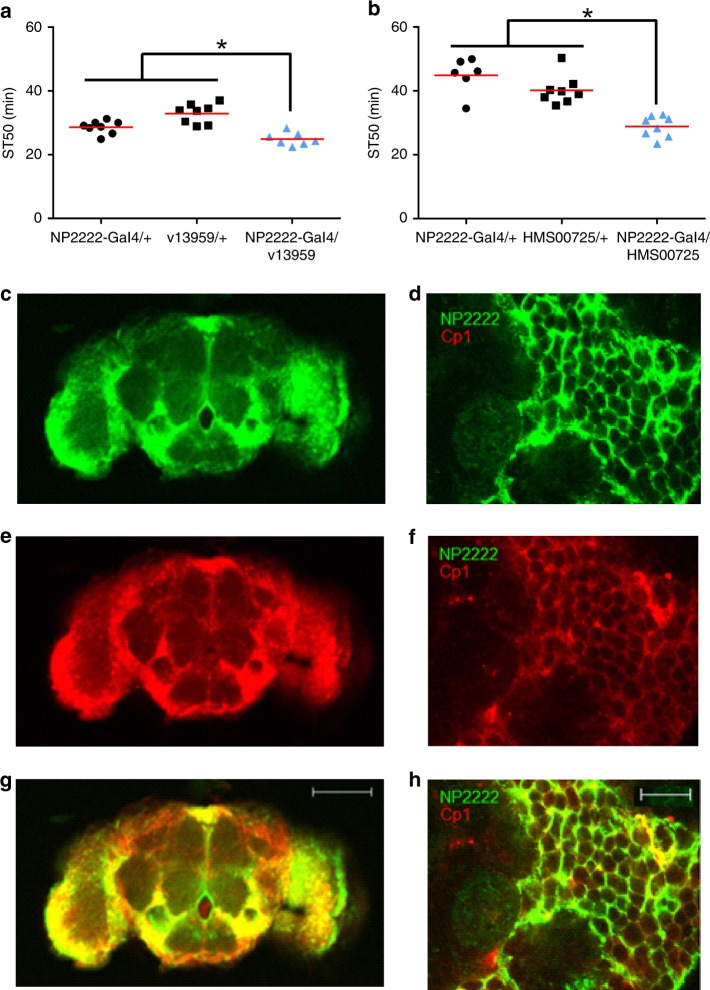
*Cp1* expression in cortex glia is required for normal ethanol sedation. **a**, **b** ST50 values were decreased in flies expressing *Cp1* RNAi transgenes in cortex glia (blue bars: *NP2222*-Gal4/v13959, panel **a**; *NP2222*-Gal4/HMS00725, panel **b**) compared to control flies containing either the cortex glia Gal4 driver (black bars: *NP2222*-Gal4/+) or the RNAi transgenes (black bars: v13959/+ or HMS00725/+) alone (individual one-way ANOVAs, *p* ≤ 0.0001; *Bonferroni’s multiple comparisons vs. controls, *p* < 0.05; *n* = 8). **c**–**h** Cp1 is expressed in cortex glia. **c**, **d** Whole-brain expression of UAS-GFP (green) driven by *NP2222*. **e**, **f** Endogenous Cp1 expression labeled red (anti-Cp1 1:250, Alexa 568 1:1000). **g**, **h** Merged image of panels **c** and **e** (**g**), and panels **d** and **f** (**h**); GFP and Cp1 co-localization is yellow. Representative images from whole brain at ×10 (**c**, **e**, **g**) and 63x oil-immersion (**d**, **f**, **h**)

Adult *Drosophila* have five CNS glial subtypes: astrocytes, ensheathing cells, cortex glia, subperineural glia, and perineural glia^12^. To address the possibility that *Cp1* influences alcohol sedation by functioning within one or more specific glial subtypes, we determined whether expression of *Cp1* RNAi transgenes in individual glial subtypes (via a series of Gal4 drivers) altered alcohol sedation sensitivity. Flies expressing *Cp1* RNAi transgenes (v13959 and HMS00725) in cortex glia (via *NP2222*-Gal4^24^ or CtxGlia Split-Gal4^23^) had decreased ST50 values compared to control flies with the Gal4 and RNAi transgenes alone (*NP2222*-Gal4: Fig. 3a, b; CtxGlia Split-Gal4: Supplementary Fig. 6a, b). Flies expressing the v110619 RNAi transgene in cortex glia (via-*NP2222*-Gal4) had inconsistent results (Supplementary Fig. 1b). Additionally, ST50 values were not altered by expression *Cp1* RNAi (v13959) in the four other CNS glial subtypes (astrocytes, ensheathing cells, subperineural glia, and perineural glia via *Alrm*-Gal4^12^, *TIFR*-Gal4^24^, *mz0709*-Gal4^24^, *Gli*-Gal4^52^ and *Indy*-Gal4^11^) (Supplementary Table 1). The simplest interpretation of these data is that *Cp1* influences alcohol sedation by functioning in cortex glia.

We used whole-brain immunofluorescence to determine if Cp1 is expressed in adult *Drosophila* cortex glia. Utilizing flies that constitutively express mCD8::GFP in cortex glia via *NP2222*-Gal4, we found that Cp1 immunofluorescence colocalized with GFP (Fig. 3c–e). When quantified using Volocity™ 3D image analysis software, >60% of the red and green pixels overlapped (average Pearson’s correlation = 0.622; *n* = 6). This result indicated that endogenous Cp1 is expressed in cortex glia, consistent with a role for Cp1 in acute alcohol sedation sensitivity.

### *Cp1* in rapid tolerance development

**Fig. 4:**
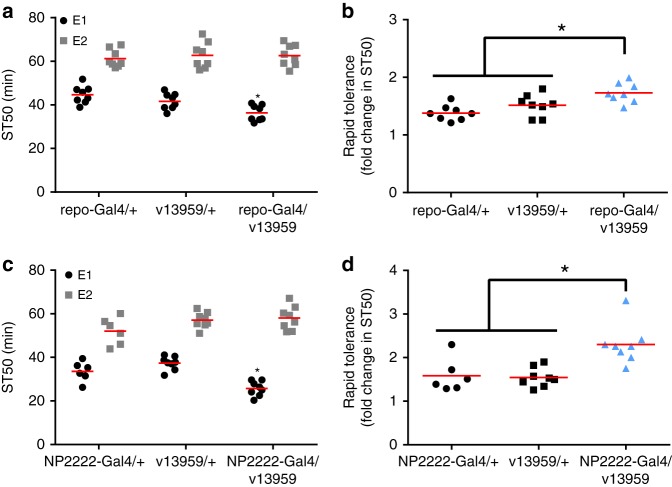
*Cp1* in rapid tolerance development. **a** ST50 values from the first (E1) and second (E2) ethanol exposure when *Cp1* is knocked down in all CNS glia. Compared to controls (*repo*-Gal4/+ and v13959/+), expression of *Cp1* RNAi in CNS glia (*repo*-Gal4/v13959) decreased ST50 values during E1, but not during E2 (two-way ANOVA; genotype, n.s.; ethanol exposure, *p* < 0.0001; interaction, *p* = 0.015; *Bonferroni’s multiple comparisons vs. controls for each ethanol exposure, *p* < 0.05; *n* = 8). **b** Development of rapid tolerance (fold change in ST50 from E1 to E2) quantified from the data in **a**. Expression of *Cp1* RNAi in glia (blue bar: *repo*-Gal4/v13959) increased rapid tolerance development compared to controls (black bars: *repo*-Gal4/+, v13959/+) (one-way ANOVA, *p* = 0.0014; *Bonferroni’s multiple comparisons vs. controls, *p* < 0.05; *n* = 8). **c** ST50 values from the first (E1) and second (E2) ethanol exposure when *Cp1* is knocked down in cortex glia. Compared to controls (*NP2222*-Gal4/+ and v13959/+), expression of *Cp1* RNAi in cortex glia (*NP2222*-Gal4/v13959) decreased ST50 during E1, but not during E2 (two-way ANOVA; ethanol exposure, *p* < 0.0001; genotype, *p* = 0.0034; interaction, *p* = 0.0001; *Bonferroni’s multiple comparisons vs. controls for each ethanol exposure, *p* < 0.05; *n* = 8). **d** Development of rapid tolerance (fold change in ST50 from E1 to E2) quantified from the data in **c**. Expression of *Cp1* RNAi in cortex glia (blue bar: *NP2222*-Gal4/v13959) increased rapid tolerance development compared to controls (black bars: *NP2222*-Gal4/+, v13959/+) (one-way ANOVA, *p* = 0.0009; *Bonferroni’s multiple comparisons vs. controls, *p* < 0.05; *n* = 8)

Flies develop rapid tolerance to alcohol, defined as increased ST50 values during a second alcohol exposure after recovering from a first alcohol exposure^53^. To determine whether *Cp1* influences this aspect of alcohol behavior through its function in CNS glia, we expressed *Cp1* RNAi transgenes in all glia (via *repo*-Gal4) and then assessed rapid tolerance development. As anticipated, pan-glial knockdown of *Cp1* via RNAi transgene v13959 significantly decreased ST50 values during the first ethanol exposure (black bars, E1) as compared to Gal4 and RNAi transgene alone controls (Fig. 4a). In contrast, ST50 values during the second alcohol exposure (gray bars, E2) were not affected by* Cp1* knockdown (Fig. 4a). When quantified as the ratio between the second and first ST50 values^53^, flies with *Cp1* knocked down in all glia had an increase in the development of rapid tolerance compared to controls (Fig. 4b). As we found during the first alcohol exposure (Fig. 1c), there was no effect of knocking-down *Cp1* in all glia on internal alcohol levels during the second alcohol exposure (One-way ANOVA, *p* = 0.85, *n* = 6). Knockdown of *Cp1* specifically in cortex glia (via *NP2222*-Gal4) also reduced ST50 values during the first, but not the second, alcohol exposure (Fig. 4c), leading to an apparent increase in development of rapid tolerance compared to controls (Fig. 4d). Given that *Cp1* knockdown does not significantly impact ST50 values during the second alcohol exposure (Fig. 4a, c), the most parsimonious interpretation of these data is that the increased development of rapid tolerance is likely a mathematical product of the enhanced sensitivity to alcohol during the first exposure. We therefore did not further investigate the potential role of *Cp1* in rapid tolerance.

### *Cp1* knockdown in adult glia alters alcohol sedation

**Fig. 5:**
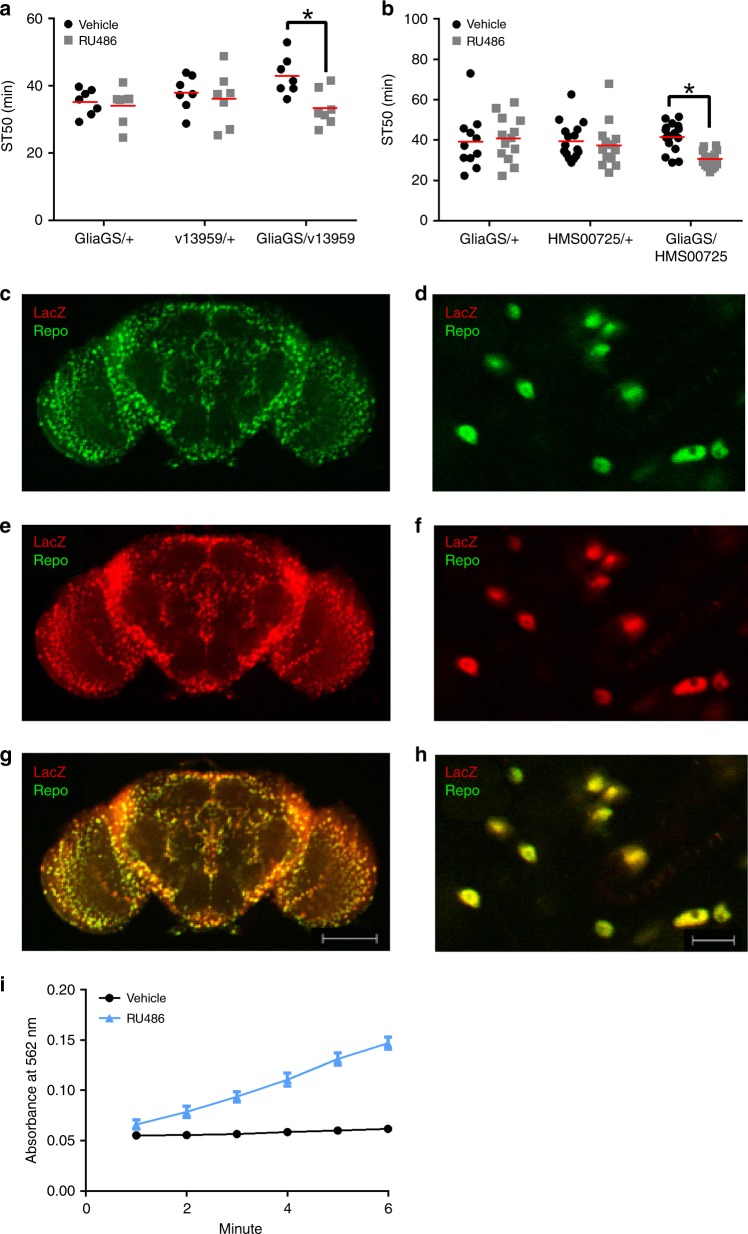
*Cp1* knockdown in CNS glia during adulthood increased ethanol sedation sensitivity. **a**, **b** Compared to vehicle, treatment with 1 mM RU486 for 6 days decreased ST50 values in flies with the GliaGS driver and a *Cp1* RNAi transgene (GliaGS/v13959, panel **a**; GliaGS/HMS00725, panel **b**), but not in control flies with either GliaGS or an RNAi transgene alone (panel **a**: two-way ANOVA; RU486, *p* = 0.0247; genotype, n.s.; interaction, n.s.; *Bonferroni’s multiple comparisons between vehicle and RU486, *p* < 0.05; *n* = 8; panel **b**: two-way ANOVA; RU486, n.s.; genotype, n.s.; interaction, *p* = 0.0411; *Bonferroni’s multiple comparisons between vehicle and RU486, *p* < 0.05; *n* = 16). **c**–**h** GliaGS drives expression in CNS glia during adulthood. GliaGS/*LacZ* flies were fed 1 mM RU486 for 6 days prior to brain dissection and immunolabeling. **c**, **d** Endogenous repo expression (green) indicating CNS glia (anti-repo 1:100, Alexa 488 1:1000) (**e**, **f**) GliaGS-driven LacZ expression labeled red (anti-LacZ 1:500, Alexa 568 1:1000) (**g**, **h**) merged images of panels **c** and **e** (**g**) and panels **d** and **f** (**h**); yellow indicates co-localization of repo and LacZ. Representative images from ×10, scale bar = 100 µm (**c**, **e**, **g**) and 63x oil, scale bar = 10 µm (**d**, **f**, **h**). **i** Treatment of GliaGS/UAS-LacZ flies with 1 mM RU486 for 6 days induced β-galactosidase activity in whole-fly extracts (blue line) compared to vehicle control (black line)

CNS glia play important roles during both development^16,54,55^ and adulthood^16,19,39^. To determine if *Cp1* expression in glia during adulthood is important for alcohol sedation, we used the steroid-inducible GeneSwitch system^56^. Flies with both the GliaGS driver and a *Cp1* RNAi transgene, and control flies with either GliaGS or the RNAi transgene alone, were reared to adulthood in the absence of the steroid mifepristone (RU486) and then switched to food medium containing steroid (RU486) or vehicle for 6 days. This RU486 feeding regimen did not alter ST50 values in control flies (see GliaGS/+ control in Fig. 5a, b). In this experimental design, the *Cp1* RNAi transgene should be induced in RU486-exposed adult flies harboring both a GeneSwitch Gal4 driver and an RNAi transgene^56^, thereby allowing *Cp1* knockdown during adulthood. Compared to vehicle control animals of the same genotype, GliaGS/v13959, GliaGS/HMS00725, and GliaGS/v110619 flies fed RU486 had decreased ST50 values (v13959 and HMS00725: Fig. 5a, b; v110619: Supplementary Fig. 1c). Exposure to RU486 in flies with either the GliaGS alone (GliaGS/+) or the *Cp1* RNAi transgenes alone (v13959/+, HMS00725/+ and v110619/+) did not alter ST50 values (Fig. 5a, b and Supplementary Fig. 1c). Manipulation of *Cp1* in glia during adulthood was therefore sufficient to increase alcohol sedation. Interestingly, substantial overexpression of *Cp1*^41^ (87%, quantified via immunofluorescence) in glia during adulthood did not change ST50 values (Supplementary Fig. 7). These results are consistent with a model in which endogenous, physiological levels of Cp1 in glia are required and sufficient for normal alcohol sedation in flies, suggesting that biologically relevant levels of Cp1 in glia contribute to alcohol behaviors.

To confirm that GliaGS-induced UAS transgenes are expressed in glia, we performed whole-brain immunofluorescence on flies with GliaGS driving induced expression of a UAS-*LacZ* transgene (i.e., GliaGS/*LacZ* flies fed RU486 for 6 days). Endogenous repo expression (a marker for glia, Fig. 5c) and induced LacZ expression driven by GliaGS (Fig. 5d) were detected in broad patterns throughout the fly brain that overlapped considerably (Fig. 5e). When quantified using Volocity™ 3D image analysis software, >90% of the red and green pixels overlapped (average Pearson’s correlation = 0.915; *n* = 7). Additionally, we found that the RU486 exposure regimen used in our behavioral studies (Fig. 5a, b) increased UAS-*LacZ* expression in whole fly extracts (Fig. 5f), demonstrating RU486-induced UAS-transgene expression. Taken together, these data indicate that GliaGS expresses UAS transgenes in glia in response to RU486 treatment, and therefore that *Cp1* influences alcohol sedation by functioning during adulthood in CNS glia.

## Discussion

Our understanding of the molecular-genetic basis for alcohol-related behavior in *Drosophila* and other model systems is based primarily on the results of studies that have focused on neuronal genes and mechanisms^7^. The nervous systems of flies and mammals also contain numerous classes of glia with conserved cellular-molecular activities. Given that mammalian glia respond to alcohol administration^57–59^, that rodent astrocytes in the nucleus acumbens influence the motivation for alcohol consumption, and that surface glia influence alcohol sedation and tolerance in flies^9–11^, it is likely that glia play direct—but underappreciated—roles in behavioral responses to alcohol.

Here, we used tissue-specific RNAi-mediated knockdown and trans-species rescue of RNAi to explore this possibility. Pan-glial *Cp1* knockdown via RNAi increased alcohol sedation. Expression of an orthologous gene, *Drosophila pseudoobscura GA25021*, in all glia rescued the alcohol sedation phenotype due to knockdown of endogenous *Cp1*. Taken together, these results indicate that *Cp1* expression in glia regulates alcohol sedation. Additionally, our studies found that *Cp1* expression specifically in cortex glia, and probably not other CNS glia, influences alcohol sedation. The magnitude and direction of change in alcohol sedation observed when *Cp1* was knocked down in all glia vs. only cortex glia were similar, suggesting that cortex glia are the principal cell type in which *Cp1* functions to regulate alcohol sedation. These results reveal a previously unidentified role for *Cp1* and cortex glia in *Drosophila* alcohol sedation. Thus, perineural glia^10,11^ and cortex glia (our results) influence behavioral responses to alcohol in *Drosophila*.

Glia have prominent roles in nervous system development in flies^23,54^. Major changes in *Drosophila* nervous system development—in response to altered glial cell function—could, in principle, alter alcohol sedation sensitivity. Our data indicate that manipulation of *Cp1* in glia during adulthood is sufficient to alter alcohol sedation in flies. Our findings are therefore consistent with a model in which *Cp1* dynamically regulates adult glial cell function, and those changes in adult glial cell function influence the response of the nervous system to alcohol.

To date, a few studies have investigated the role of *Drosophila* cortex glia in behavior. One study suggests that innexin2 expression in cortex glia is required for normal sleep patterns^60^, and two studies have indicated that cortex glia function contributes to seizures^22,61^. Additionally, cortex glia morphology influences larval locomotor behaviors^23^. The results reported here add to the emerging literature on cortex glia and behavior by showing that cortex glia, via *Cp1* function, influence alcohol sedation. It could be important to explore the role of cortex glia, in conjunction with *Cp1* and other candidate pathways, in behavioral responses to other drugs of abuse.

*Cp1* knockdown in glia, specifically cortex glia, appeared to enhance alcohol rapid tolerance development. However, glial *Cp1* knockdown influenced sedation during the first exposure to alcohol only. These results suggest that *Cp1* function in glia selectively influences alcohol sedation during an initial exposure to the drug and any interpretations regarding the role of *Cp1* in rapid tolerance should be made with considerable caution. Importantly, though, since *Cp1* knockdown in glia did not influence alcohol sedation during a second alcohol exposure or alter locomotor abilities in the absence of alcohol (Supplementary Fig. 8), it seems unlikely that the initial sedation sensitivity of flies with *Cp1* knockdown in glia is related to global sluggishness, a lack of overall behavioral fitness, or other experimental artifacts. We therefore posit that glial *Cp1* plays a direct role in response of the central nervous system to alcohol.

Cp1 cleaves, and thereby activates, the transcription factor cut^42^. Additionally, the protein crammer binds to and inactivates the Cp1 protein^36^. We consequently predicted that altered expression of cut or crammer might alter sedation sensitivity. Surprisingly, constitutive expression of RNAi against *cut* or *crammer* in cortex glia or adult-specific expression of RNAi in all glia failed to substantively alter alcohol sedation (Supplementary Figs. 9 and 10). Although additional follow-up studies would be required to formally rule out a role for cut or crammer in Cp1-dependent alcohol sedation, our data suggest that *Cp1* influences alcohol sedation independently of these two known genes.

Cp1 is structurally and functionally homologous to mammalian Cathepsin L^43^. Cathepsins are powerful hydrolytic cysteine proteases and are inactively stored in the lysosomes of most tissues in mammalian cells^62^. When released from lysosomes in their active form, they play roles in many physiological processes^62^. Although Cathepsin L has not been directly implicated in alcohol-related behaviors in mammals, Cathepsin L contributes to alcohol-induced cellular and/or organ damage. For example, Cathepsin L mediates alcohol-induced pancreatic damage and alcoholic liver fibrosis^63,64^. Following alcohol administration, Cathepsin L is activated in pancreatic lysosomes^63,65^ and down-regulated in the cellular matrix in the liver^64^, contributing to disease pathologies. However, it is unlikely that altered alcohol sedation in *Cp1* knockdown flies is caused by over-all cathepsin-related glial cell damage because flies with *Cp1* knockdown have normal locomotor responses in the absence of alcohol (Supplementary Fig. 8) and *Cp1* knockdown selectively alters alcohol sedation during a first, but not a second, exposure to the drug (Fig. 4a). Additionally, *Cp1* overexpression in all glia during adulthood does not alter alcohol sedation (Supplementary Fig. 7). Although our results do not rule out the possibility that *Cp1* is involved in glial cell damage, they do suggest that alcohol sedation sensitivity in *Cp1* knockdown animals is unrelated to cellular damage that potentially may be occurring.

Cathepsin L also functions in secretory vesicles as a proneuropeptide processing^66^. Cathepsin L knockdown resulted in an 80–90% reduction of Neuropeptide Y (NPY) production in mammals^66^. Interestingly, NPY is synthesized in glia during development and adulthood in mammals. During adulthood, glial NPY is postulated to provide trophic support to neurons^67^. Mammalian NPY is homologous to *Drosophila* Neuropeptide F (NPF), which influences alcohol sedation in *Drosophila*^68,69^. While a role for Cp1 in NPF maturation in flies is possible, it seems unlikely that glial *Cp1* influences alcohol sedation via processing of NPF. When NPF synthesis was ablated in all NPF-producing cells, alcohol sedation was blunted^69^, whereas knockdown of *Cp1*, which would also be predicted to decrease NPF production, increased alcohol sedation in our studies. These contradictory results make it very unlikely that *Cp1* and NPF are working in conjunction to mediate alcohol sedation in *Drosophila*. Thus, additional studies, potentially involving approaches grounded in proteomics, are required to begin elucidating the molecular mechanisms involved in *Cp1*-dependent modulation of alcohol sedation in flies.

In summary, our results suggest a previously unidentified and potentially direct role for *Drosophila* glia in alcohol-related behaviors and that *Cp1* represents a functional entry point for further understanding of cortex glial mechanisms that underlie alcohol sedation. Given that *Drosophila* Cp1 is orthologous to mammalian Cathepsin L, and that fly cortex glia are functionally similar to mammalian protoplasmic astrocytes, our findings have the potential to be translatable to mammalian systems. Our findings also raise the possibility that glial cysteine proteinases might mediate behavioral responses to other drugs of abuse in both flies and mammals.

## Methods

### Fly husbandry

All flies were reared under standard conditions^53^. Flies were grown on food medium containing 10% sucrose, 3.3% cornmeal, 2% yeast, 1% agar, 0.2% Tegosept, and antibiotics (0.1 g/L ampicillin, 0.02 g/L tetracycline, 0.125 g/L chloramphenicol) with active dry yeast on top in 6-ounce polypropylene *Drosophila* bottles (Fisher Scientific, Hampton, NH). Flies were housed in an environmental chamber kept at 25 °C and 60% relative humidity with a 12-h light/dark cycle. All comparisons between groups were based on studies with flies that were grown, handled, and tested side by side.

### Fly stocks

UAS-RNAi transgenic strains to manipulate *Cp1* expression were obtained from commercial/public resources: v13959 and v110619, Vienna *Drosophila* Resource Center (VDRC), Vienna, Austria; HMS00725 (stock number 32932) and EY05806 (stock number 15957), Bloomington *Drosophila* Stock Center (BDSC), Bloomington, IN. HMS00725, marked with *y*+, was backcrossed to a *w*^*1*^*y*^*1*^ strain (stock number 1495, BDSC) for seven generations to normalize the genetic background. UAS-RNAi strains targeting *crammer* (v22751 and v22752) and *cut* (v4138 and v5687) were obtained from the VDRC. A w^1118^ reference stock from the VDRC (stock number 60000) was used to control the genetic background of all flies obtained from this stock center. The UAS-*LacZ* transgenic strain used to validate GliaGS-induced expression was obtained from BDSC (stock number 6452). Gal4 drivers were obtained from the indicated sources: *repo*-Gal4 (BDSC, stock number 7415), *elav*-Gal4 (BDSC, stock number 8760), *Alrm3*-Gal4 and *mz0709*-Gal4 (Marc Freeman, Oregon Health Sciences University, Portland, OR, USA), *NP2222*-Gal4 and *TIFR*-Gal4 (Mary Logan, Oregon Health Sciences University, Portland, OR, USA), CtxGlia Split-Gal4 (Jaeda Coutinho-Budd, University of Vermont, Burlington, VT, USA), *Indy*-Gal4 (Fred Wolf, University of California–Merced, Merced, CA, USA) and *Gli*-Gal4 (Doris Kretzschmar, Oregon Health Sciences University, Portland, OR, USA). All Gal4 stocks (marked with mini-*w*) were backcrossed to our standard reference strain, *w*[A] (*w*^*1118*^ in an isogenic background; BDSC, stock number 5905) for seven generations to normalize the genetic background. The steroid-inducible Gal4 driver, GliaGS, was obtained from the BDSC (stock number 59929, GeneSwitch ID 7293–1). The *NP2222*-Gal4 strain that constitutively expressed mCD8::GFP was provided by Mary Logan (Oregon Health Sciences University, Portland, OR, USA).

### Ethanol sedation sensitivity and rapid tolerance

One day before behavioral studies, adult flies (1–4-days-old) were placed under light CO_2_ anesthesia and sorted for sex. Eleven adult female flies were placed into fresh non-yeasted food vials (standard food medium without active dry yeast on top). Flies recovered in food vials stored upside down (food side up) overnight at 25 °C and 60% relative humidity. Each vial of flies corresponded to *n* = 1; up to 24 vials were tested in each single ethanol sedation experiment.

Ethanol sedation studies were performed at 23–25 °C and 55–65% relative humidity under standard office lighting. Flies, after a 1–2 h acclimation period in the testing room, were transferred to empty polystyrene food vials (VWR, Radnor, PA) and trapped in the vials with a cellulose acetate Flug (FlyStuff, San Diego, CA) inserted ~2 cm into each vial. The number of inactive flies was recorded for each vial (typically 0–1 flies/vial). One milliliter of 85% ethanol (made fresh weekly) was added to each Flug, and the vials were immediately sealed with a silicone stopper. Once every 6 min, each vial was tapped gently on a table three  times and the number of sedated flies (i.e., still on the bottom of the vial) was recorded 30 s later. The ethanol sedation experiments were terminated when all flies were sedated, typically after 60–90 min. The percentage of active flies was calculated for each vial at each time point, and the time required for 50% of the flies in each vial to become sedated (sedation time 50, ST50) was interpolated from sigmoidal curve fits using Excel (Microsoft, Redwood, WA)^53,70^.

Rapid tolerance to ethanol was assessed as the change in sensitivity to ethanol sedation due to a prior exposure to the drug. Flies were tested for ethanol sedation during a first ethanol exposure as described above (E1), returned to food vials to recover for 4 h, and then tested for ethanol sedation during a second ethanol exposure (E2)^53^. The development of rapid tolerance was quantitated as the ratio between the ST50 during E2 and the ST50 during E1.

### Internal ethanol

Flies were exposed to vapor from 85% ethanol as described for measuring ethanol sedation^71^. After exposure to ethanol vapor for a duration equivalent to the ST50, flies were transferred to 1.5 mL snap-cap tubes and frozen at −80 °C. Frozen flies were homogenized in 200 µL ice-cold ddH_2_0 and then centrifuged at 14,000 rpm at 4 °C for 20 min. The internal ethanol concentration of the supernatant was determined using Alcohol Reagent Set (Pointe Scientific Inc., Canton, MI) according to the manufacturer’s instructions.

### Trans-species rescue of the *Cp1* RNAi in glia

FlyBase and NCBI were used to determine that *D.melanogaster* *Cp1* and *D. pseudoobscura* *GA25021* were orthologous. Fly stocks that express *D. pseudoobscura* *GA25021* under UAS control were created via standard P-element-mediated transgenesis using pUAST^51^. The *D. pseudoobscura* *GA25021* cDNA was cloned into the pUAST vector by GenScript (Piscataway, NJ, USA) and injected in w[A], our standard lab stock, by Rainbow Transgenic Flies (Camarillo, CA, USA). We mapped the independent UAS-*GA25021* insertions to autosomes. Flies constitutively expressing the HMS00725 *Cp1* RNAi transgene in all glia via *repo*-Gal4 were generated through standard crosses.

### Quantitative real-time PCR

mRNA expression was assessed via quantitative real-time PCR (qRT-PCR)^72^. Approximately 400 fly heads per *n* were isolated. Total RNA was isolated using TRIZOL and was reverse transcribed via oligo (dT) primers and Superscript II reverse transcriptase (Invitrogen; Carlsbad, CA, USA). qRT-PCR was performed using an Applied Biosystems Fast 7500 system with SYBR Green PCR master mix (Quanta Biosciences; Beverly, MA, UAS) and run in triplicate. Each qRT-PCR experiment was performed with independent RNA isolations and cDNA syntheses, and normalized to *actin5C*. Primers used (forward/reverse) were as follows: *Cp1*, 5′- CTCATGTGACGCTGCCCAAATC-3′/5′- CCAGCACAGGCGCCCTC-3′; *GA25021*, 5′- GACAGCATTGATTCTTCCCCTCC-3′/5′- GTGTGCCATTCCTCCTGGATG-3′; *actin5C*, 5′- AGCGCGGTTACTCTTTCACCAC-3′/5′- GTGGCCATCTCCTGCTCAAAGT-3′ (Fisher Scientific, Hampton, NH, USA). Primers for *Cp1* readily detected endogenous *Cp1* from *repo*-Gal4/+ flies, but this level of expression was not significantly altered in *repo*-Gal4/UAS-*GA25021* flies (see Supplementary Fig. 5). Additionally, primers for GA25021 detected a product in *repo*-Gal4/UAS-*GA25021* flies, but this signal was not altered by expression of *Cp1* RNAi HMS00725 (see Supplementary Fig. 5). These findings confirm that the *Cp1* and *GA25021* primers were specific for their intended products.

### Locomotor behavior

Flies were collected as described above for ethanol sedation. On the test day, flies were transferred to empty polystyrene food vials. The positive control group vials (Gal4/+) were vortexed for 4 min prior to the experiment. Thereafter vials were handled as described for ethanol sedation studies, except for the following changes: no ethanol was placed on the flug and no plug was used to seal the vial. The percentage of active flies was calculated for each vial at each time point.

### GeneSwitch induction

One-hundred microliters of 1 mM Mifepristone (RU486; Sigma Aldrich, St. Louis, MO) or vehicle (100% ethanol) was added to the surface of solidified food in vials and allowed to dry overnight. Flies were provided food medium topped with RU486 (induced) or vehicle (control) for 6 days total. Flies were transferred to fresh drug- or vehicle-treated food vials after 3 days.

### β-Galactosidase activity

β-Galactosidase activity was measured in whole body extracts of flies^73^. Three adult (4-day-old) female flies were homogenized in 250 µL buffer (1x PBS with 1x protease inhibitor cocktail (Sigma Aldrich, St. Louis, MO)). An additional 500 µL of extraction buffer was added, the extracts were vortexed and then centrifuged at 14,000 rpm for 5 min at room temperature. One-hundred microliters of the resulting supernatant was added to 900 µL of 1 mM chlorophenol red-β-d-galactopyranoside (Sigma Aldrich, St. Louis, MO). β-galactosidase activity was observed as the change in absorbance at 562 nm over 6 min in a Ultraspec 2000 spectrophotometer (Pharmacia Biotech, Piscataway, NJ).

### Whole-brain imaging and immunodetection

Whole brains from adult (4-day-old) female flies were dissected in 0.3% Phosphate buffer Triton X-100 (PBT) under a dissecting microscope. Dissected brains were fixed in 0.5 mL snap-cap tubes containing 4% paraformaldehyde on ice and then for 20 min at room temperature on a tube rotator. Brains were then washed with 0.3% PBT and blocked with 5% normalized goat serum (NGS). Primary antibodies diluted in 5% NGS were added and brains were placed on a tube rotator at 4 °C for 36–48 h. Brains were washed with 0.3% PBT and exposed to the secondary antibodies diluted in 5% NGS at 4 °C for 36–48 h. Brains were then washed with 0.3% PBT and mounted onto glass slides in SlowFade mounting medium (Invitrogen, Carlsbad, CA)^74^.

The following primary antibodies at the indicated concentrations from the listed sources were used: polyclonal guinea pig anti-cp1 (1:250; donated from Patrick Dolph, Dartmouth College, NH); monoclonal mouse anti-repo (1:100, Developmental Studies Hybridoma Bank, Iowa City, IA); polyclonal rabbit anti-LacZ (1:25, Fisher Scientific). The following secondary antibodies were used: goat anti-guinea pig Alexa 568, rabbit anti-mouse Alexa 488 and chicken anti-rabbit Alexa 647 (all at 1:1000; ThermoFisher, Waltham, MA).

All images were collected using a Zeiss LSM 510 multi-photon microscope (Carl Zeiss Microscopy, LLC, Thornwood, NY) or a Zeiss LSM 700 confocal microscope (Carl Zeiss Microscopy, LLC, Thornwood, NY) housed in the VCU Department of Anatomy and Neurobiology Microscope Facility. Confocal images using a pin hole of 1 Airy disc unit and Nyquist sampling were collected were collected from each adult brain. Images were taken with a 10x objective with a numerical aperture of 0.3 or a 63x oil-immersion objective with a numerical aperture of 1.4. The gain and offset values were kept constant for all images compared within an experiment.

All images taken on the Zeiss LSM 510 multi-photon microscope were processed using Zeiss LSM Image Browser Version 4,2,0,121 and Inkscape 0.92 was used to adjust image orientation. All images taken on the Zeiss LSM 700 confocal microscope were processed using Zeiss Zen 2.3. Co-localization between glia (via endogenous repo expression) and LacZ was quantified using Volocity™ 3D Image Analysis Software version 6.3. All thresholds were automatically set and Pearson Correlation was reported. Mean pixel intensity of Z-stacks was quantified using ImageJ (NIH, Bethesda, MA, USA).

### Statistics and reproducibility

All statistical analyses (one-way ANOVA, two-way ANOVA, and Bonferroni’s multiple comparison tests) were performed with Prism 6.04 (GraphPad Software, San Diego, CA, USA). Numerical data are mean ± S.E.M.

The researcher was blinded to all groups in experiments whenever possible. All behavioral experiments were performed twice to ensure all data presented in the manuscript is reproducible. All molecular experiments utilized multiple individual samples to account for natural variations and ensure reproducibility.

### Reporting summary

Further information on research design is available in the Nature Research Reporting Summary linked to this article.

## Supplementary information

Supplementary Information

Reporting Summary

## Data Availability

The authors declare that all data supporting the findings of this study are available within the article and its supplementary information files. Requests of any additional information or data that support the findings of this study are available from the corresponding author upon reasonable request.

## References

[1] American Psychiatric, A. *Diagnostic and Statistical Manual of Mental Disorders*. 5 edn, (American Psychiatric Publishing, Washington, D.C., 2013).

[2] Mokdad AH, Marks JS, Stroup DF, Gerberding JL (2004). Actual causes of death in the United States, 2000. JAMA.

[3] Edenberg HJ, Foroud T (2013). Genetics and alcoholism. Nat. Rev. Gastroenterol. Hepatol..

[4] Barry Colleen L., Epstein Andrew J., Fiellin David A., Fraenkel Liana, Busch Susan H. (2016). Estimating demand for primary care-based treatment for substance and alcohol use disorders. Addiction.

[5] Sacks JJ, Gonzales KR, Bouchery EE, Tomedi LE, Brewer RD (2015). 2010 National and state costs of excessive alcohol consumption. Am. J. Prev. Med..

[6] Schuckit MA (1994). Low level of response to alcohol as a predictor of future alcoholism. Am. J. psychiatry.

[7] Grotewiel M, Bettinger JC (2015). Drosophila and Caenorhabditis elegans as discovery platforms for genes involved in human alcohol use disorder. Alcohol., Clin. Exp. Res..

[8] Park A, Ghezzi A, Wijesekera TP, Atkinson NS (2017). Genetics and genomics of alcohol responses in Drosophila. Neuropharmacology.

[9] Bull C (2014). Rat nucleus accumbens core astrocytes modulate reward and the motivation to self-administer ethanol after abstinence. Neuropsychopharmacol.: Off. Publ. Am. Coll. Neuropsychopharmacology.

[10] Bainton RJ (2005). Moody encodes two GPCRs that regulate cocaine behaviors and blood-brain barrier permeability in Drosophila. Cell.

[11] Parkhurst SJ (2018). Perineurial barrier glia physically respond to alcohol in an Akap200-dependent manner to promote tolerance. Cell Rep..

[12] Freeman Marc R. (2015). DrosophilaCentral Nervous System Glia. Cold Spring Harbor Perspectives in Biology.

[13] Freeman MR, Delrow J, Kim J, Johnson E, Doe CQ (2003). Unwrapping glial biology: Gcm target genes regulating glial development, diversification, and function. Neuron.

[14] Ou J, Gao Z, Song L, Ho MS (2016). Analysis of glial distribution in drosophila adult brains. Neurosci. Bull..

[15] Ma Z, Stork T, Bergles DE, Freeman MR (2016). Neuromodulators signal through astrocytes to alter neural circuit activity and behaviour. Nature.

[16] Logan MA (2017). Glial contributions to neuronal health and disease: new insights from Drosophila. Curr. Opin. Neurobiol..

[17] Limmer S, Weiler A, Volkenhoff A, Babatz F, Klambt C (2014). The Drosophila blood-brain barrier: development and function of a glial endothelium. Front. Neurosci..

[18] Volkenhoff A (2015). Glial glycolysis is essential for neuronal survival in Drosophila. Cell Metab..

[19] Kremer MC, Jung C, Batelli S, Rubin GM, Gaul U (2017). The glia of the adult Drosophila nervous system. Glia.

[20] Awasaki T, Lai SL, Ito K, Lee T (2008). Organization and postembryonic development of glial cells in the adult central brain of Drosophila. J. Neurosci.: Off. J. Soc. Neurosci..

[21] Pereanu W, Spindler S, Cruz L, Hartenstein V (2007). Tracheal development in the Drosophila brain is constrained by glial cells. Dev. Biol..

[22] Melom JE, Littleton JT (2013). Mutation of a NCKX eliminates glial microdomain calcium oscillations and enhances seizure susceptibility. J. Neurosci.: Off. J. Soc. Neurosci..

[23] Coutinho-Budd JC, Sheehan AE, Freeman MR (2017). The secreted neurotrophin Spatzle 3 promotes glial morphogenesis and supports neuronal survival and function. Genes Dev..

[24] Doherty J, Logan MA, Tasdemir OE, Freeman MR (2009). Ensheathing glia function as phagocytes in the adult Drosophila brain. J. Neurosci.: Off. J. Soc. Neurosci..

[25] Liu H (2014). Astrocyte-like glial cells physiologically regulate olfactory processing through the modification of ORN-PN synaptic strength in Drosophila. Eur. J. Neurosci..

[26] MacNamee SE (2016). Astrocytic glutamate transport regulates a Drosophila CNS synapse that lacks astrocyte ensheathment. J. Comp. Neurol..

[27] Stork T, Sheehan A, Tasdemir-Yilmaz OE, Freeman MR (2014). Neuron-glia interactions through the Heartless FGF receptor signaling pathway mediate morphogenesis of Drosophila astrocytes. Neuron.

[28] Zhang, Y. V., Ormerod, K. G. & Littleton, J. T. Astrocyte Ca2+ influx negatively regulates neuronal activity. *eNeuro* **4**, 10.1523/ENEURO.0340-1516.2017 (2017). 10.1523/ENEURO.0340-16.2017 PMC5348542 28303263

[29] Peco E (2016). Drosophila astrocytes cover specific territories of the CNS neuropil and are instructed to differentiate by Prospero, a key effector of Notch. Dev. (Camb., Engl.).

[30] Otto N (2018). The sulfite oxidase Shopper controls neuronal activity by regulating glutamate homeostasis in Drosophila ensheathing glia. Nat. Commun..

[31] Dutta S, Rieche F, Eckl N, Duch C, Kretzschmar D (2016). Glial expression of Swiss cheese (SWS), the Drosophila orthologue of neuropathy target esterase (NTE), is required for neuronal ensheathment and function. Dis. Models Mech..

[32] Kazama H, Yaksi E, Wilson RI (2011). Cell death triggers olfactory circuit plasticity via glial signaling in Drosophila. J. Neurosci.: Off. J. Soc. Neurosci..

[33] Hindle SJ (2017). Evolutionarily conserved roles for blood-brain barrier xenobiotic transporters in endogenous steroid partitioning and behavior. Cell Rep..

[34] Danjo R, Kawasaki F, Ordway RW (2011). A tripartite synapse model in Drosophila. PloS ONE.

[35] Stork T (2008). Organization and function of the blood-brain barrier in Drosophila. J. Neurosci.: Off. J. Soc. Neurosci..

[36] Deshapriya RM (2007). Drosophila CTLA-2-like protein (D/CTLA-2) inhibits cysteine proteinase 1 (CP1), a cathepsin L-like enzyme. Zool. Sci..

[37] Ng FS (2016). TRAP-seq profiling and RNAi-based genetic screens identify conserved glial genes required for adult Drosophila behavior. Front. Mol. Neurosci..

[38] Ng FS, Jackson FR (2015). The ROP vesicle release factor is required in adult Drosophila glia for normal circadian behavior. Front. Cell. Neurosci..

[39] Zwarts L, Van Eijs F, Callaerts P (2015). Glia in Drosophila behavior. J. Comp. Physiol. A, Neuroethol., Sens., neural, Behav. Physiol..

[40] Dong Q, Brenneman B, Fields C, Srivastava A (2015). A Cathepsin-L is required for invasive behavior during Air Sac Primordium development in Drosophila melanogaster. FEBS Lett..

[41] Kinser RD, Dolph PJ (2012). Cathepsin proteases mediate photoreceptor cell degeneration in Drosophila. Neurobiol. Dis..

[42] Lyons GR, Andersen RO, Abdi K, Song WS, Kuo CT (2014). Cysteine proteinase-1 and cut protein isoform control dendritic innervation of two distinct sensory fields by a single neuron. Cell Rep..

[43] Tryselius Y, Hultmark D (1997). Cysteine proteinase 1 (CP1), a cathepsin L-like enzyme expressed in the Drosophila melanogaster haemocyte cell line mbn-2. Insect Mol. Biol..

[44] Gray YH, Sved JA, Preston CR, Engels WR (1998). Structure and associated mutational effects of the cysteine proteinase (CP1) gene of Drosophila melanogaster. Insect Mol. Biol..

[45] Mohamed MM, Sloane BF (2006). Cysteine cathepsins: multifunctional enzymes in cancer. Nat. Rev. Cancer.

[46] Schwartz MK (1995). Tissue cathepsins as tumor markers. Clin. Chim. acta; Int. J. Clin. Chem..

[47] Cermak S (2016). Loss of cathepsin B and L leads to lysosomal dysfunction, NPC-like cholesterol sequestration and accumulation of the key Alzheimer’s proteins. PLoS ONE.

[48] Dietzl G (2007). A genome-wide transgenic RNAi library for conditional gene inactivation in Drosophila. Nature.

[49] Perkins LA (2015). The transgenic RNAi project at Harvard Medical School: resources and validation. Genetics.

[50] Gramates LS (2017). FlyBase at 25: looking to the future. Nucl. acids Res..

[51] Kondo S, Booker M, Perrimon N (2009). Cross-species RNAi rescue platform in Drosophila melanogaster. Genetics.

[52] Schmidt I (2012). Kinesin heavy chain function in Drosophila glial cells controls neuronal activity. J. Neurosci.: Off. J. Soc. Neurosci..

[53] Chan RF (2014). Contrasting influences of Drosophila white/mini-white on ethanol sensitivity in two different behavioral assays. Alcohol., Clin. Exp. Res..

[54] Fernandes VM, Chen Z, Rossi AM, Zipfel J, Desplan C (2017). Glia relay differentiation cues to coordinate neuronal development in Drosophila. Sci. (New Y., N. Y.).

[55] Isaacman-Beck J, Clandinin TR (2017). Glia put visual map in sync. Sci. (New Y., N. Y.).

[56] Nicholson L (2008). Spatial and temporal control of gene expression in Drosophila using the inducible GeneSwitch GAL4 system. I. Screen for larval nervous system drivers. Genetics.

[57] Adermark L, Bowers MS (2016). Disentangling the role of astrocytes in alcohol use disorder. Alcohol., Clin. Exp. Res..

[58] Blednov YA (2012). Neuroimmune regulation of alcohol consumption: behavioral validation of genes obtained from genomic studies. Addict. Biol..

[59] Miguel-Hidalgo J, Shoyama Y, Wanzo V (2009). Infusion of gliotoxins or a gap junction blocker in the prelimbic cortex increases alcohol preference in Wistar rats. J. Psychopharmacol. (Oxf., Engl.).

[60] Farca Luna AJ, Perier M, Seugnet L (2017). Amyloid precursor protein in Drosophila glia regulates sleep and genes involved in glutamate recycling. J. Neurosci.: Off. J. Soc. Neurosci..

[61] Kunduri G (2018). Defective cortex glia plasma membrane structure underlies light-induced epilepsy in cpes mutants. Proc. Natl Acad. Sci. USA.

[62] Conus S, Simon HU (2008). Cathepsins: key modulators of cell death and inflammatory responses. Biochem. Pharmacol..

[63] Kanbak G (2012). Preventive role of gallic acid on alcohol dependent and cysteine protease-mediated pancreas injury. Mol. Biol. Rep..

[64] Mir RA, Chauhan SS (2011). Down regulation of a matrix degrading cysteine protease cathepsin L, by acetaldehyde: role of C/EBPalpha. PLoS ONE.

[65] Thrower EC, Gorelick FS, Husain SZ (2010). Molecular and cellular mechanisms of pancreatic injury. Curr. Opin. Gastroenterol..

[66] Funkelstein L, Beinfeld M, Minokadeh A, Zadina J, Hook V (2010). Unique biological function of cathepsin L in secretory vesicles for biosynthesis of neuropeptides. Neuropeptides.

[67] Ubink R, Calza L, Hokfelt T (2003). ‘Neuro’-peptides in glia: focus on NPY and galanin. Trends Neurosci..

[68] Shohat-Ophir G, Kaun KR, Azanchi R, Mohammed H, Heberlein U (2012). Sexual deprivation increases ethanol intake in Drosophila. Sci. (New Y., N. Y.).

[69] Wen T, Parrish CA, Xu D, Wu Q, Shen P (2005). Drosophila neuropeptide F and its receptor, NPFR1, define a signaling pathway that acutely modulates alcohol sensitivity. Proc. Natl Acad. Sci. USA.

[70] Sandhu, S., Kollah, A. P., Lewellyn, L., Chan, R. F. & Grotewiel, M. An inexpensive, scalable behavioral assay for measuring ethanol sedation sensitivity and rapid tolerance in Drosophila. *J. Vis. Exp.: JoVE* . 10.3791/52676 (2015). 10.3791/52676 PMC4423423 25939022

[71] Bhandari P, Kendler KS, Bettinger JC, Davies AG, Grotewiel M (2009). An assay for evoked locomotor behavior in Drosophila reveals a role for integrins in ethanol sensitivity and rapid ethanol tolerance. Alcohol. Clin. Exp. Res..

[72] Jones MA (2009). A forward genetic screen in Drosophila implicates insulin signaling in age-related locomotor impairment. Exp. Gerontol..

[73] Martin I (2009). Sod2 knockdown in the musculature has whole-organism consequences in Drosophila. Free Radic. Biol. Med..

[74] Wu JS, Luo L (2006). A protocol for dissecting Drosophila melanogaster brains for live imaging or immunostaining. Nat. Protoc..

