# Supplementary material for: Alcohol sedation in adult Drosophila is regulated by Cysteine proteinase-1 in cortex glia
Source: Commun Biol. 2019 Jul 3;2:252. doi: 10.1038/s42003-019-0492-5 (PMC6610072; doi:10.1038/s42003-019-0492-5)
Supplement: Supplementary file 1 — Supplementary Information [file 42003_2019_492_MOESM1_ESM.pdf]

Supplementary Figures

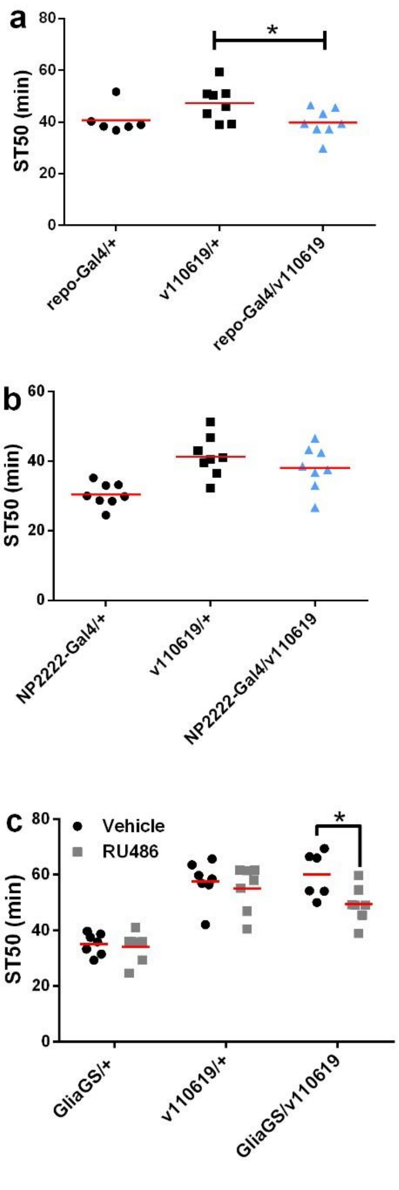

Supplementary Figure 1. Ethanol sedation sensitivity in flies expressing *Cp1* RNAi v110619 in glia. (**a**) ST50 values were influenced by overall genotype and reduced in flies expressing the *Cp1* RNAi transgene v110619 in all CNS glia (blue triangles: *repo*-Gal4/v110619) compared to one control group (black squares: v1101619/+), but not the other control group (black circles, repo-Gal4/+) (one-way ANOVA, p = 0.0465; *Bonferroni's multiple

comparisons, v110619/+ vs. *repo*-Gal4/v110619, $p < 0.05$; $n = 8$). (**b**) ST50 values were influenced by overall genotype (one-way ANOVA, $p = 0.0015$, $n=8$) but were not detectably different in planned comparisons between flies expressing *Cp1* RNAi v110619 in cortex glia (blue triangles: *NP2222*-Gal4/v110619) and control flies containing the NP2222-Gal4 alone (black circles) or the v110619 RNAi transgene alone (black squares) (**c**) Expression of *Cp1* RNAi in CNS glia during adulthood increased ethanol sedation sensitivity. Compared to vehicle-treated controls, treatment with 1 mM RU486 for 6 days decreased ST50 values in flies with the GliaGS driver and *Cp1* RNAi transgene (GliaGS/v110619), but not in control flies with either GliaGS or the RNAi transgene alone (two-way ANOVA; RU486, $p = 0.0341$; genotype, $p < 0.0001$; interaction, n.s.; *Bonferroni's multiple comparisons between vehicle and RU486, $p < 0.05$; $n = 8$).

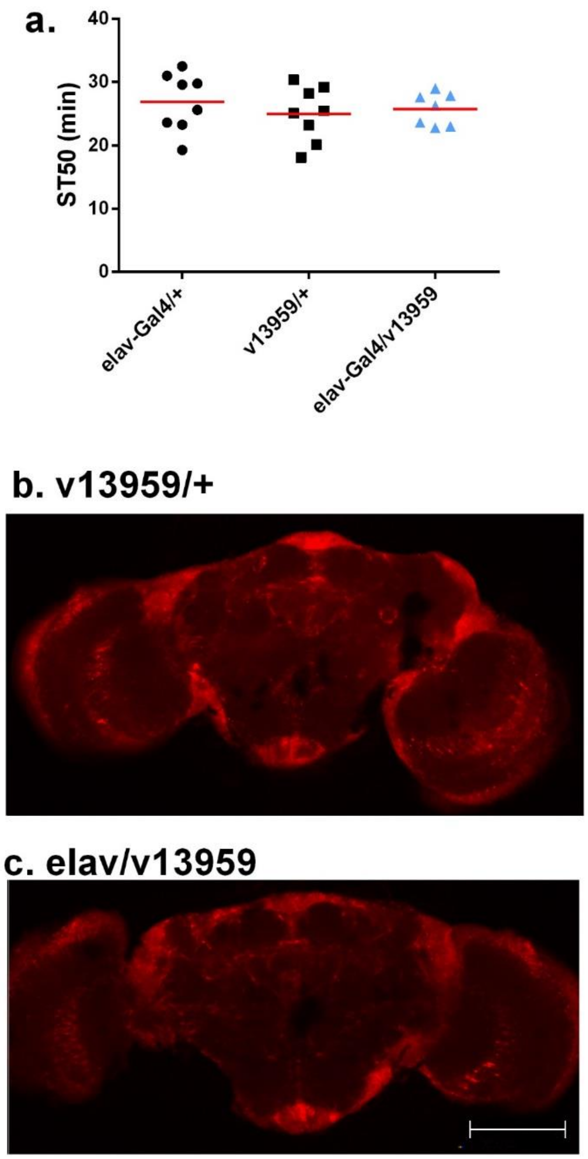

Supplementary Figure 2. Expression of *Cp1* RNAi in neurons did not influence ethanol sedation sensitivity. (a) ST50 values were not changed in flies expressing the *Cp1* RNAi transgene v13959 in neurons (blue triangles: *elav*-Gal4/v13959) compared to control flies with either *elav*-Gal4 alone (black circles: elav-Gal4/+) or the RNAi transgene alone (black squares: v13959/+) (one-way ANOVA, $p = 0.6508$; $n = 8$). **(b, c)** Whole mount brains immunolabeled for Cp1 detection (Anti-Cp1 1:250, Alexa 568 1:1000). Cp1 fluorescence was reduced 29% in flies with the *Cp1* RNAi v13959 expressed pan-neuronally (via *elav*-Gal4) (panel c) compared to flies

that had the *Cp1* RNAi transgene alone (panel b). Microscope settings were optimized for v13959/+ brains. Mean fluorescence intensity was calculated using Image J, n = 5. 10X, scale bar = 100 µm, representative images.

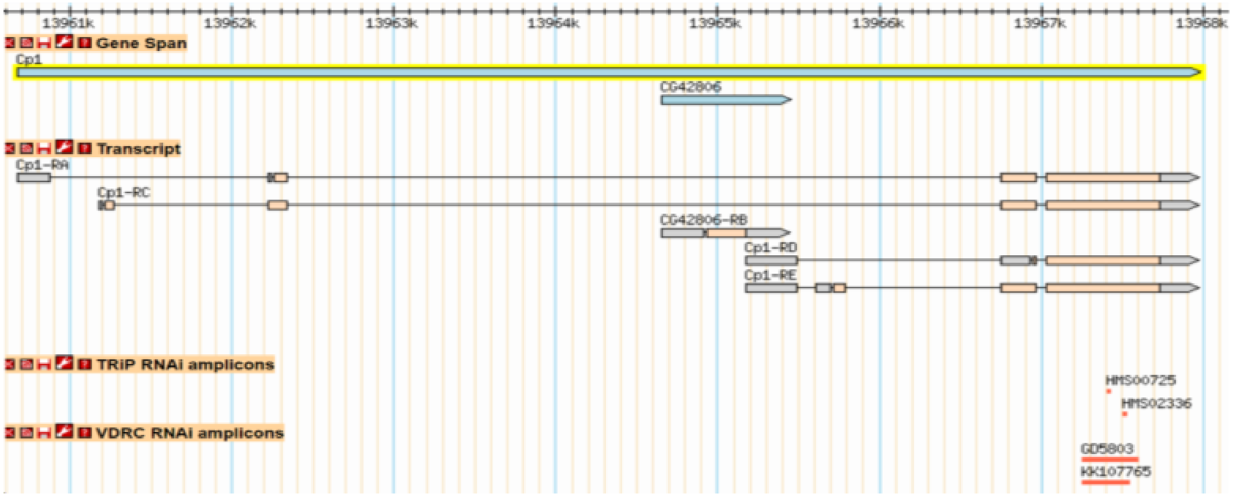

Supplementary Figure 3. The *Cp1* region. Transcription is from left to right. Complementary sequences for UAS-*Cp1* RNAi transgenes (HMS00725 and v13959 (labeled as GD5803) and v110619 (labeled as KK107765)) are shown below the predicted transcripts. All 3 RNAis are predicted to cleave all 4 *Cp1* transcripts. Image taken from the FlyBase genome browser (www.FlyBase.org)[1].

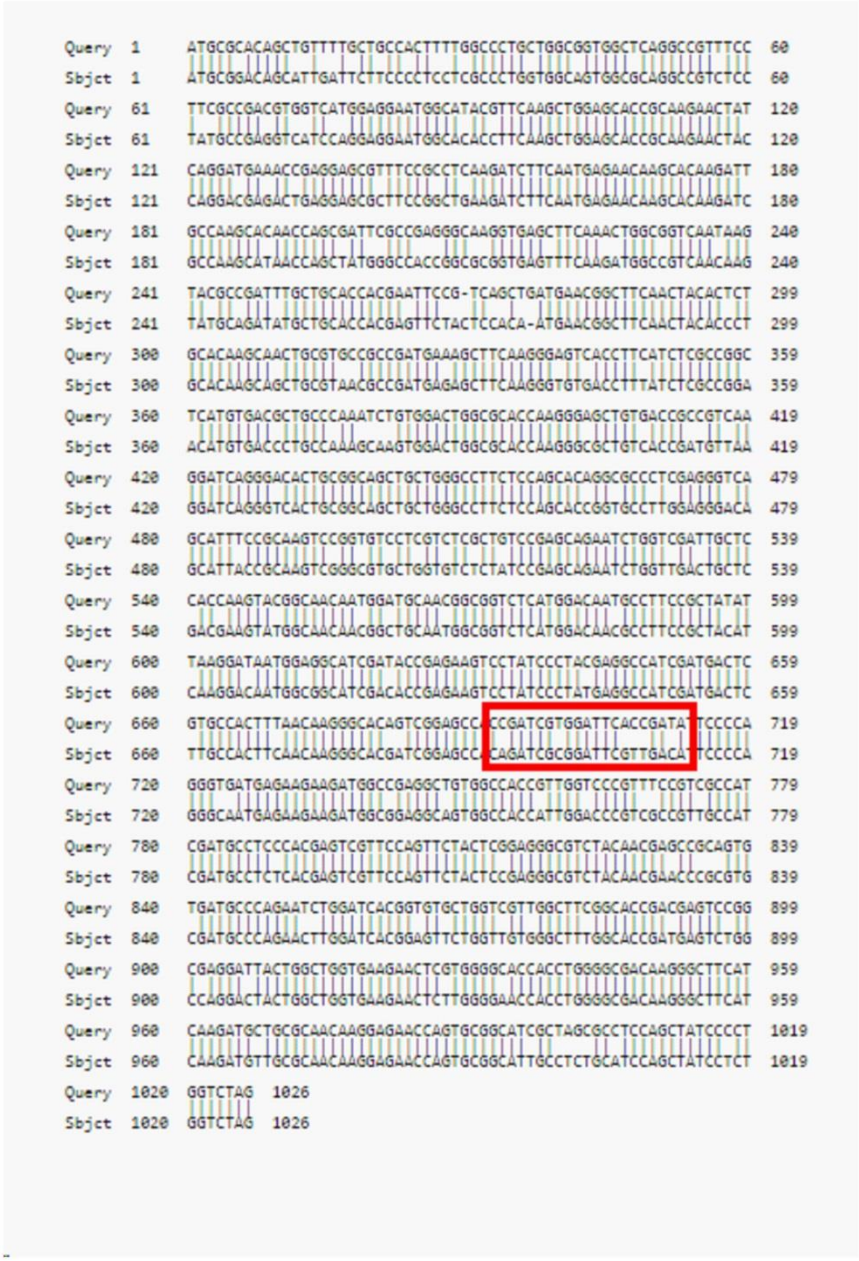

Supplementary Figure 4. ***HMS00725 siRNA target region on Drosophila melanogaster Cp1 and Drosophila pseudoobscura GA25021 alignment.*** The *Drosophila melanogaster* Cp1 transcript is “Query 1” and the *Drosophila pseudoobscura* GA25021 transcript is “Sbjct 1”. The HMS00725 siRNA target sequence on the Cp1 transcript is boxed in red. Comparing this region between Cp1 and GA25021, 6 base pairs are mismatched.

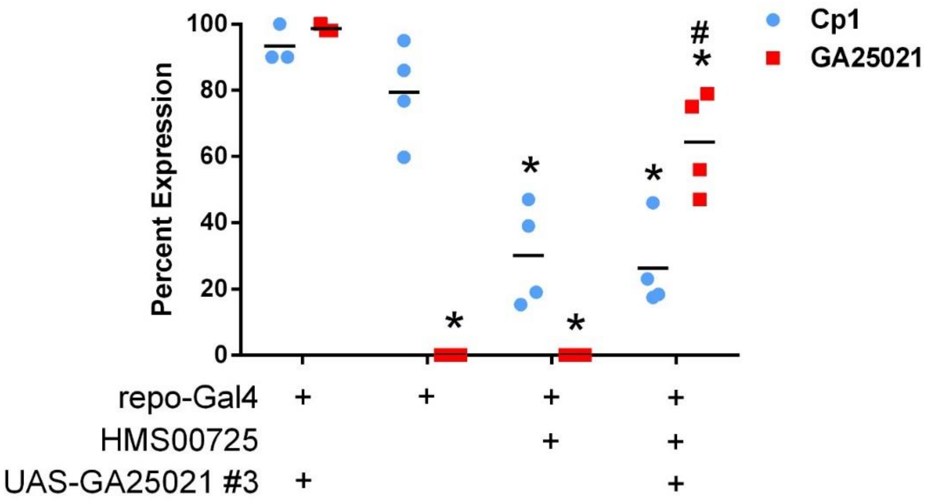

Supplementary Figure 5. ***qRT-PCR validation that HMS00725 reduces Cp1 mRNA and UAS-GA25021 #3 induces GA25021 mRNA expression.*** *repo*-Gal4/UAS-GA25021 #3 flies served as mRNA product level controls since they were the only genotype tested that had endogenous Cp1 and induced GA25021 in glia. Thus, all mRNA levels are normalized to *repo*-Gal4/UAS-GA25021 flies. *repo*-Gal4/+ flies had indistinguishable Cp1 expression, but GA25021 expression was undetectable. *repo*-Gal4;HMS00725/+ flies had significantly decreased levels of Cp1 with undetectable GA25021. *repo*-Gal4;HMS00725/UAS-GA25021 #3 flies had significantly decreased levels of Cp1 and GA25021. However, Cp1 mRNA levels in *repo*-Gal4;HMS00725/+ and *repo*-Gal4;HMS00725/UAS-GA25021 #3 flies were indistinguishable. Additionally, GA25021 mRNA levels were significantly increased in *repo*-Gal4;HMS00725/UAS0GA2521 #3 flies compared to *repo*-Gal4/+ and *repo*-Gal4;HMS00725/+ flies (two-way ANOVA; genotype, $p < 0.0001$; gene detected, $p < 0.0005$; interaction, $p < 0.0001$.; Bonferroni's multiple comparisons between genotypes, $p < 0.05$; * represents statistical comparisons to *repo*-Gal4/UAS-GA25021 #3; # represents statistical comparisons to *repo*-Gal4;HMS00725/+; n = 4).

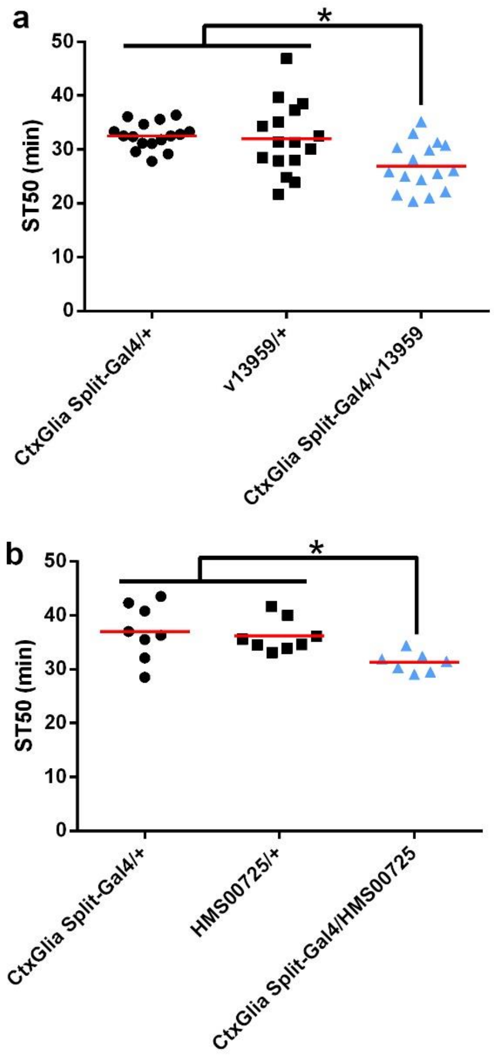

Supplementary Figure 6. Cp1 expression in cortex glia is required for normal ethanol sedation. (**a, b**) ST50 values were decreased in flies expressing *Cp1* RNAi transgenes in cortex glia (blue triangles: CtxGlia Split-Gal4/v13959, panel a; CtxGlia Split-Gal4/HMS00725, panel b) compared to control flies containing either the cortex glia Gal4 driver (black circles: CtxGlia Split-Gal4/+) or the RNAi transgenes (black squares: v13959/+ or HMS00725/+) alone (Panel a: one-way ANOVA, $p = 0.0029$; *Bonferroni's multiple comparisons vs controls, $p < 0.05$; $n = 16$; Panel b: one-way ANOVA, $p = 0.0156$; Bonferroni's multiple comparisons vs controls, $p < 0.05$; $n = 8$).

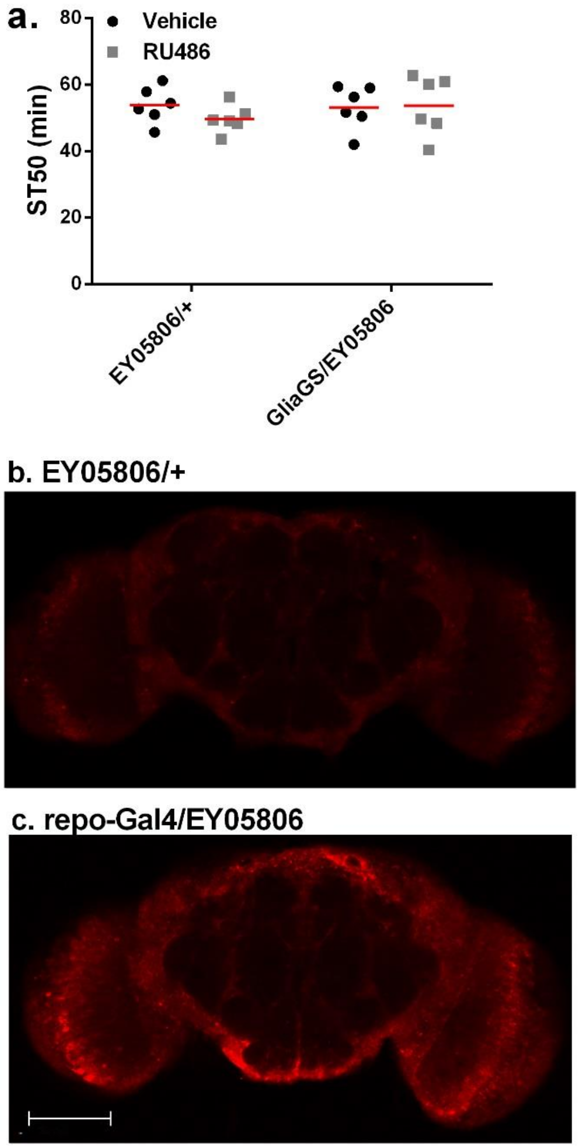

Supplementary Figure 7. Over-expression of Cp1 in CNS glia during adulthood did not alter ethanol sedation sensitivity. (a) Over-expression of Cp1 in CNS glia during adulthood via the EPgy2 transposon insertion EY05806 did not change ethanol sedation sensitivity. Compared to vehicle-treated controls, treatment with 1 mM RU486 for 6 days did not alter ST50 values in flies with the GliaGS driver and a UAS-*Cp1* transgene (GliaGS/EY05806). Control flies with the UAS-*Cp1* transgene alone also had no change in ST50 between vehicle and RU486 treatment (EY05806/+) (two-way ANOVA; RU486, n.s.; genotype, n.s.; interaction, n.s; all

Bonferroni's multiple comparisons between vehicle and RU486, $p > 0.05$; $n = 8$). **(b, c)** Whole mount brains immunolabeled for Cp1 (Anti-Cp1 1:250, Alexa 568 1:1000). Brains from flies with EY05806 and *repo*-Gal4 had increased fluorescence (89%) compared to brains from flies with EY05806 alone. Microscope settings were optimized for *repo*-Gal4/EY05806 brains to avoid over-saturation in the image analysis. Mean fluorescence intensity was calculated using Image J, n = 5-6. 10X, scale bar = 100 µm, representative images.

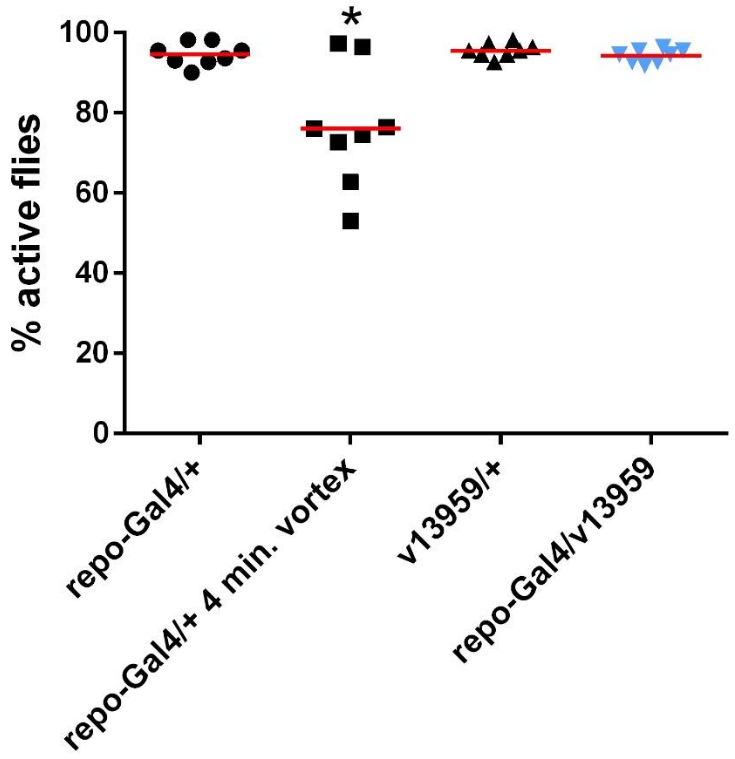

Supplementary Figure 8. Expression of *Cp1* RNAi in CNS glia did not alter locomotion. Expression of the *Cp1* RNAi transgene v13959 in CNS glia (via *repo*-Gal4) did not alter the percentage of active flies compared to controls with either *repo*-Gal4 or the RNAi transgene alone. Vortexing the *repo*-Gal4 control for 4 minutes reduced the percentage of active flies compared to the other groups (one-way ANOVA, $p < 0.001$; * Bonferronni's multiple comparison, $p < 0.05$; $n = 8$)

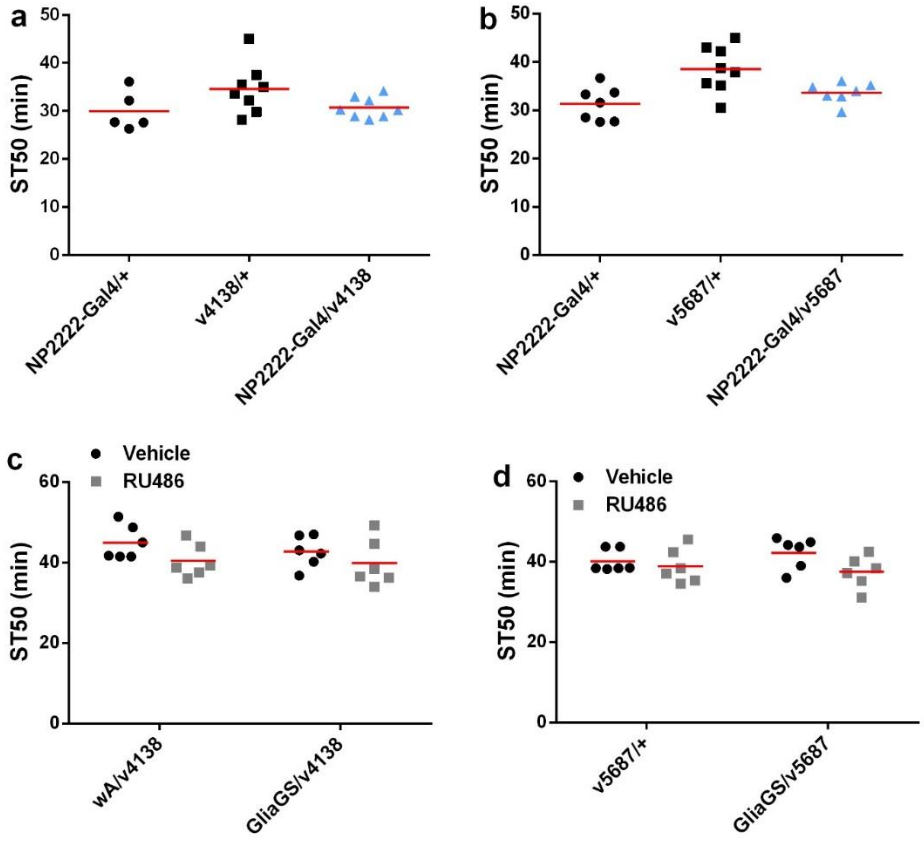

Supplementary Figure 9. Expression of *cut* RNAi in cortex glia and CNS glia during adulthood did not alter ethanol sedation sensitivity. (**a, b**) ST50 values were not changed in flies expressing *cut* RNAi transgenes in cortex glia (blue triangles: *NP2222*-Gal4/v4138, panel a; blue triangles: *NP2222*-Gal4/v5687, panel b) compared to control flies containing either the cortex glia Gal4 driver (black circles: *NP2222*-Gal4/+) or the RNAi transgenes (black squares: v4138/+ or v5687/+) alone (Panel a: one-way ANOVA, $p > 0.05$; $n = 8$; Panel b: one-way ANOVA, $p = 0.0041$; all Bonferroni's multiple comparisons vs controls, $p > 0.05$; $n = 8$). (**c, d**) Expression of *cut* RNAi in CNS glia during adulthood did not alter ethanol sedation sensitivity. Compared to vehicle-treated controls, treatment with 1 mM RU486 for 6 days did not change ST50 values in flies with the GliaGS driver and a *cut* RNAi transgene (GliaGS/v4138, panel c; GliaGS/v5687, panel d). Control flies with the RNAi transgene alone also had no change in ST50 between vehicle and RU486 treatment (individual two-way ANOVAs; RU486, n.s.;

genotype, n.s.; interaction, n.s.; all Bonferroni's multiple comparisons between vehicle and RU486, $p > 0.05$; $n = 8$).

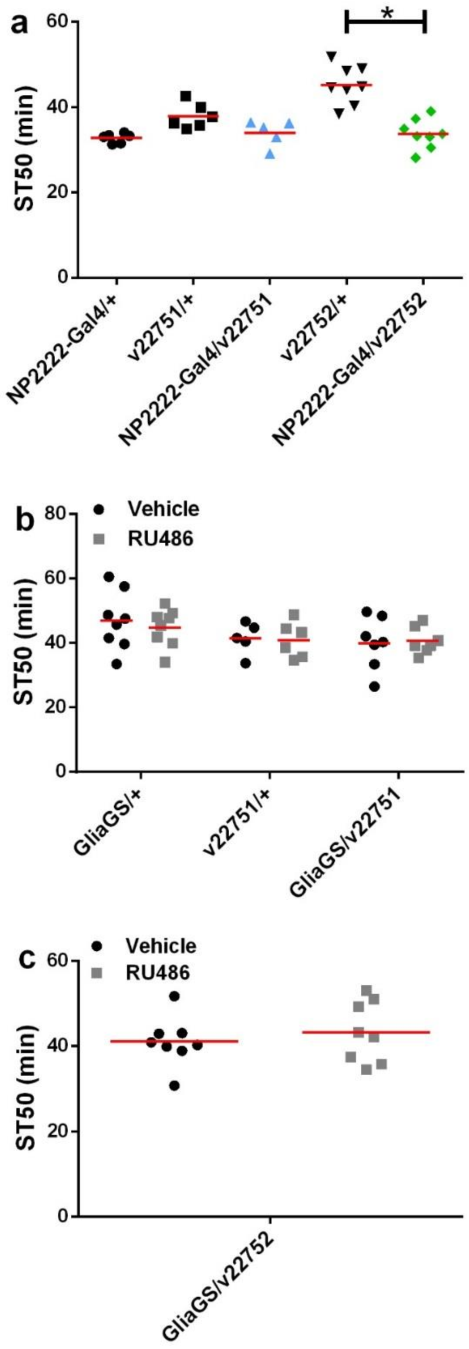

Supplemental Figure 10. Expression of *crammer* RNAi in cortex glia and CNS glia during adulthood did not alter ethanol sedation sensitivity. (**a**) ST50 values were significantly decreased between flies expressing the *crammer* RNAi transgene v22752 in cortex glia (blue triangles: *NP2222*-Gal4/v22752) compared to the RNAi alone (v22752/+) control (one-way

ANOVA, $p < 0.0001$; *Bonferroni's multiple comparisons vs controls, $p > 0.05$; $n = 8$) However, ST50 values were not changed in flies expressing *crammer* RNAi transgenes in cortex glia (blue triangles diamonds: *NP2222*-Gal4/v22751; green diamonds: *NP2222*-Gal4/v22752) compared to control flies containing either the cortex glia Gal4 driver (black circles: *NP2222*-Gal4/+) or the appropriate RNAi transgene (black squares: v22751/+) alone (one-way ANOVA, $p < 0.0001$; *Bonferroni's multiple comparisons vs controls, $p < 0.05$; $n = 8$). (**b, c**) Expression of *crammer* RNAi in CNS glia during adulthood does not alter ethanol sedation sensitivity. (**b**) Compared to vehicle-treated controls, treatment with 1 mM RU486 for 6 days did not alter ST50 values in flies with the GliaGS driver and the *crammer* RNAi transgene v22751 (GliaGS/v22751). Control flies with either GliaGS or the RNAi transgene alone also had no differences in ST50 between vehicle and RU486 treatment (two-way ANOVA; interaction, n.s.; genotype, $p = 0.0302$; RU486, n.s.; all Bonferroni's multiple comparisons between vehicle and RU486, $p > 0.05$; $n = 3$-8). (**c**) Compared to vehicle-treated controls, treatment with 1 mM RU486 for 6 days did not alter ST50 values in flies with the GliaGS driver and the *crammer* RNAi transgene v22751 (GliaGS/v22751). (t-test, $p > 0.05$; $n = 8$).

Supplementary Tables

Glial subtype (Gal4)	Genotype	ST50	ANOVA	Multiple comparisons (to Gal4/RNAi)
Ensheathing cells (*TIFR*-Gal4)	Gal4/+	36.86 ± 3.833 (7)	F (2,18) = 3.893, p = 0.0393	p > 0.05
	RNAi/+	31.10 ± 3.624 (7)$		p > 0.05
	Gal4/RNAi	35.23 ± 4.437 (7)		n/a
Subperineural glia (*Gli*-Gal4)	Gal4/+	33.67 ± 3.185 (7)	F (2,18) = 1.564, p = 0.236	n/a
	RNAi/+	31.10 ± 3.624 (7)$		n/a
	Gal4/RNAi	31.66 ± 3.185 (7)		n/a
Astrocytes (*Alrm*-Gal4)	Gal4/+	26.6 ± 1.241 (8)	F (2,21) = 0.381 , p = 0.687	n/a
	RNAi/+	25.96 ± 1.066 (8)#		n/a
	Gal4/RNAi	25.66 ± 3.430 (8)		n/a
Ensheathing cells (*mz0709*-Gal4)	Gal4/+	32.56 ± 4.084 (8)	F (2,21) = 10.85, p = 0.0006	p < 0.05
	RNAi/+	25.96 ± 1.066 (8)#		p > 0.05
	Gal4/RNAi	26.26 ± 3.592 (8)		n/a
Perineural glia (*Indy*-Gal4)	Gal4/+	34.6 ± 1.262 (8)	F(2,21) = 0.1931, p = 0.825	n/a
	RNAi/+	33.34 ± 1.645 (8)		n/a
	Gal4/RNAi	34.03 ± 1.382 (8)		n/a

Supplementary Table 1. Expression of the *Cp1* RNAi transgene v13959 in ensheathing cells (via *TIFR*-Gal4 and *mz0709*-Gal4), subperineural glia (via *Gli*-Gal4), astrocytes (via *Alrm*-Gal4) and perineural glia (via *Indy*-Gal4) did not consistently alter alcohol sedation compared to both Gal4 driver (Gal4/+) and RNAi transgene (RNAi/+) controls. Results from individual one-way ANOVAs and (when appropriate) Bonferroni's multiple comparisons are reported. $ and # represent common RNAi/+ controls.

Supplementary References

1 Gramates, L. S. *et al.* FlyBase at 25: looking to the future. *Nucleic acids research* **45**, D663-D671, doi:10.1093/nar/gkw1016 [doi] (2017).
