# Supplementary material for: Alcohol sedation in adult Drosophila is regulated by Cysteine proteinase-1 in cortex glia
Source: Commun Biol. 2019 Jul 3;2:252. doi: 10.1038/s42003-019-0492-5 (PMC6610072; doi:10.1038/s42003-019-0492-5)
Supplement: Supplementary file 2 — Reporting Summary [file 42003_2019_492_MOESM2_ESM.pdf]

nature research

Corresponding author(s): Grotewiel, Michael COMMSBIO-18-0381B

Last updated by author(s): May 14, 2019

Reporting Summary

Nature Research wishes to improve the reproducibility of the work that we publish. This form provides structure for consistency and transparency in reporting. For further information on Nature Research policies, see Authors & Referees and the Editorial Policy Checklist.

Statistics

For all statistical analyses, confirm that the following items are present in the figure legend, table legend, main text, or Methods section.

n/a	Confirmed	
☐	☒	The exact sample size (*n*) for each experimental group/condition, given as a discrete number and unit of measurement
☐	☒	A statement on whether measurements were taken from distinct samples or whether the same sample was measured repeatedly
☐	☒	The statistical test(s) used AND whether they are one- or two-sided<br>*Only common tests should be described solely by name; describe more complex techniques in the Methods section.*
☒	☐	A description of all covariates tested
☒	☐	A description of any assumptions or corrections, such as tests of normality and adjustment for multiple comparisons
☐	☒	A full description of the statistical parameters including central tendency (e.g. means) or other basic estimates (e.g. regression coefficient) AND variation (e.g. standard deviation) or associated estimates of uncertainty (e.g. confidence intervals)
☒	☐	For null hypothesis testing, the test statistic (e.g. *F*, *t*, *r*) with confidence intervals, effect sizes, degrees of freedom and *P* value noted<br>*Give P values as exact values whenever suitable.*
☒	☐	For Bayesian analysis, information on the choice of priors and Markov chain Monte Carlo settings
☒	☐	For hierarchical and complex designs, identification of the appropriate level for tests and full reporting of outcomes
☒	☐	Estimates of effect sizes (e.g. Cohen's *d*, Pearson's *r*), indicating how they were calculated

Our web collection on statistics for biologists contains articles on many of the points above.

Software and code

Policy information about availability of computer code

Data collection	n/a
Data analysis	n/a

For manuscripts utilizing custom algorithms or software that are central to the research but not yet described in published literature, software must be made available to editors/reviewers. We strongly encourage code deposition in a community repository (e.g. GitHub). See the Nature Research guidelines for submitting code & software for further information.

Data

Policy information about availability of data

All manuscripts must include a data availability statement. This statement should provide the following information, where applicable:

- Accession codes, unique identifiers, or web links for publicly available datasets
- A list of figures that have associated raw data
- A description of any restrictions on data availability

The authors declare that all data supporting the findings of this study are available within the article and its supplementary information files. Requests of any additional information or data that support the findings of this study are available from the corresponding author upon reasonable request.

Field-specific reporting

Please select the one below that is the best fit for your research. If you are not sure, read the appropriate sections before making your selection.

☒ Life sciences ☐ Behavioural & social sciences ☐ Ecological, evolutionary & environmental sciences

Life sciences study design

All studies must disclose on these points even when the disclosure is negative.

Sample size	Previous power analyses indicate that samples of 8 are adequate for detecting changes of 20% in ST50s derived from alcohol sedation studies under the conditions used.
Data exclusions	No data were excluded.
Replication	All key findings were replicated in multiple experiments using independent fly populations.
Randomization	All behavioral tests were performed on groups of animals that had been randomized.
Blinding	Experimenters were blind to group in all behavioral studies.

Reporting for specific materials, systems and methods

We require information from authors about some types of materials, experimental systems and methods used in many studies. Here, indicate whether each material, system or method listed is relevant to your study. If you are not sure if a list item applies to your research, read the appropriate section before selecting a response.

Materials & experimental systems

n/a	Involved in the study	
☐	☒	Antibodies
☒	☐	Eukaryotic cell lines
☒	☐	Palaeontology
☐	☒	Animals and other organisms
☒	☐	Human research participants
☒	☐	Clinical data

Methods

n/a	Involved in the study	
☒	☐	ChIP-seq
☒	☐	Flow cytometry
☒	☐	MRI-based neuroimaging

Antibodies

Antibodies used	The following primary antibodies at the indicated concentrations from the listed sources were used: polyclonal guinea pig anti-cp1 (1:250; donated from Patrick Dolph, Dartmouth College, NH); monoclonal mouse anti-repo (1:100, Developmental Studies Hybridoma Bank, Iowa City, IA); polyclonal rabbit anti-LacZ (1:25, Fisher Scientific). The following secondary antibodies were used: goat anti-guinea pig Alexa 568, rabbit anti-mouse Alexa 488 and chicken anti-rabbit Alexa 647 (all at 1:1000; ThermoFisher, Waltham, MA).
Validation	Anti-cp1 was validated by RNAi studies in the article. Anti-repo was previously validated as described (http://dshb.biology.uiowa.edu/8D12-anti-Repo). Anti-lacZ was validated as described (www.fisherscientific.com).

Animals and other organisms

Policy information about studies involving animals; ARRIVE guidelines recommended for reporting animal research

Laboratory animals	Drosophila melanogaster
Wild animals	n/a
Field-collected samples	n/a
Ethics oversight	n/a

Note that full information on the approval of the study protocol must also be provided in the manuscript.
